# Arbuscular mycorrhizal fungi and *Streptomyces*: brothers in arms to shape the structure and function of the hyphosphere microbiome in the early stage of interaction

**DOI:** 10.1186/s40168-024-01811-2

**Published:** 2024-05-09

**Authors:** Zexing Jin, Feiyan Jiang, Letian Wang, Stéphane Declerck, Gu Feng, Lin Zhang

**Affiliations:** 1 State Key Laboratory of Nutrient Use and Management, College of Resources and Environmental Sciences, Key Laboratory of Plant-Soil Interactions, Ministry of Education, China Agricultural University, Beijing, 100193 China; 2 Applied Microbiology, Mycology, Earth and Life Institute, Université Catholique de Louvain, Croix du Sud 2, Bte L7.05.06, Louvain-La-Neuve, B-1348 Belgium

**Keywords:** Bacterial community, Fungi and bacteria interaction, Hyphosphere, In vitro culture, Keystone taxa, Organic P

## Abstract

**Background:**

Fungi and bacteria coexist in a wide variety of environments, and their interactions are now recognized as the norm in most agroecosystems. These microbial communities harbor keystone taxa, which facilitate connectivity between fungal and bacterial communities, influencing their composition and functions. The roots of most plants are associated with arbuscular mycorrhizal (AM) fungi, which develop dense networks of hyphae in the soil. The surface of these hyphae (called the hyphosphere) is the region where multiple interactions with microbial communities can occur, e.g., exchanging or responding to each other’s metabolites. However, the presence and importance of keystone taxa in the AM fungal hyphosphere remain largely unknown.

**Results:**

Here, we used in vitro and pot cultivation systems of AM fungi to investigate whether certain keystone bacteria were able to shape the microbial communities growing in the hyphosphere and potentially improved the fitness of the AM fungal host. Based on various AM fungi, soil leachates, and synthetic microbial communities, we found that under organic phosphorus (P) conditions, AM fungi could selectively recruit bacteria that enhanced their P nutrition and competed with less P-mobilizing bacteria. Specifically, we observed a privileged interaction between the isolate *Streptomyces* sp. D1 and AM fungi of the genus *Rhizophagus*, where (1) the carbon compounds exuded by the fungus were acquired by the bacterium which could mineralize organic P and (2) the in vitro culturable bacterial community residing on the surface of hyphae was in part regulated by *Streptomyces* sp. D1, primarily by inhibiting the bacteria with weak P-mineralizing ability, thereby enhancing AM fungi to acquire P.

**Conclusions:**

This work highlights the multi-functionality of the keystone bacteria *Streptomyces* sp. D1 in fungal-bacteria and bacterial-bacterial interactions at the hyphal surface of AM fungi.

Video Abstract

**Supplementary Information:**

The online version contains supplementary material available at 10.1186/s40168-024-01811-2.

## Background

Bacteria and fungi coexist in a wide variety of environments, and their interactions (so-called bacterial-fungal interactions (BFIs)) are crucial in the functioning of many agroecosystems, driving biogeochemical cycles and contributing to plant nutrition and health [1, 2]. Recently, co-occurrence analysis between bacteria and fungi revealed complex ecological processes such as cross-feeding, competition, and predation [3–5]. Interactions involving keystone species — defined as “highly interconnected species that drive community responses through microbe-microbe interactions” [1] — have been reported in the phyllosphere microbiome as well as in leaf litter, soils, and plants [3, 5–8].

The hyphosphere — “the microhabitat surrounding hyphal cells” [1] — also provides niches that are colonized by bacterial communities that may play key roles in hyphal growth, production of secondary metabolites, and reproduction and are therefore important in processes such as the fermentation of foods (e.g., cheese), beverages (e.g., beer), and cultivation of edible mushrooms [9–12].

In the last decade, increasing attention has been devoted to the hyphosphere of arbuscular mycorrhizal (AM) fungi, which are obligatory symbionts of plants. Given that AM fungal mycelia vastly extend in soil (from 82 to 111 m cm^−3^ in prairie and 52 to 81 m cm^−3^ in ungrazed pasture [13]) and mobilize between 4 and 20% of the total carbon (C) synthesized by plants [14, 15], they can be considered as key players in ecosystems, enabling bacterial activity (e.g., solubilization of organic phosphorus (P)) by excretion of compounds used as nutrients or acting as signals at their surface [16]. This hyphosphere interface thus represents a huge biological market for C and nutrients (especially P) with trade-offs between AM fungi and bacteria [17]. This is important for the function of AM fungi, as they have little capacity to utilize organic nutrients and thus must cooperate with bacteria in the hyphosphere [18–20]. For instance, recent studies have reported that some bacteria (e.g., *Rahnella aquatilis* — a phosphate solubilizing bacterium (PSB)) are stimulated at the hyphal surface of AM fungi, further allowing AM fungi to obtain P from organic P substrates [16]. Interestingly, AM fungi can specifically enrich/select some bacterial groups and reject others at their hyphal surface [21]. These selected bacteria might constitute the keystone candidate taxa of the microbial communities evolving in the hyphosphere. However, it is still unknown whether such bacterial taxa may shape hyphosphere communities, impacting fitness of AM fungi. Current knowledge on the AM fungal hyphosphere is still limited, since the study of BFI in this niche requires specific methodologies [20, 22]. Among them, the in vitro cultivation of AM fungi on root organs or on whole plants has allowed the production of a large amount of AM hyphae in a root-free and contaminant-free compartment.

Here, we used the in vitro cultivation system on carrot root organs to investigate BFI in the AM fungal hyphosphere, with phytate as the unique P source. The results were then confirmed with synthetic communities of bacteria (SynComs) under pot cultivation systems in the greenhouse. Three hypotheses were specifically addressed: (1) AM fungi recruited specific bacteria from a community of in vitro culturable bacteria as keystone taxa; (2) the selected keystone taxa had strong abilities to mobilize organic P, exchanging benefit with AM fungi, e.g., acquiring C source; and (3) the keystone taxa could inhibit other bacteria from competition with the AM fungus for P source, therefore influencing the microbial community evolving at the hyphal surface.

## Results

### The genus *Streptomyces* was a keystone taxon in the in vitro culturable bacterial community of the hyphosphere of AM fungi

**Fig. 1:**
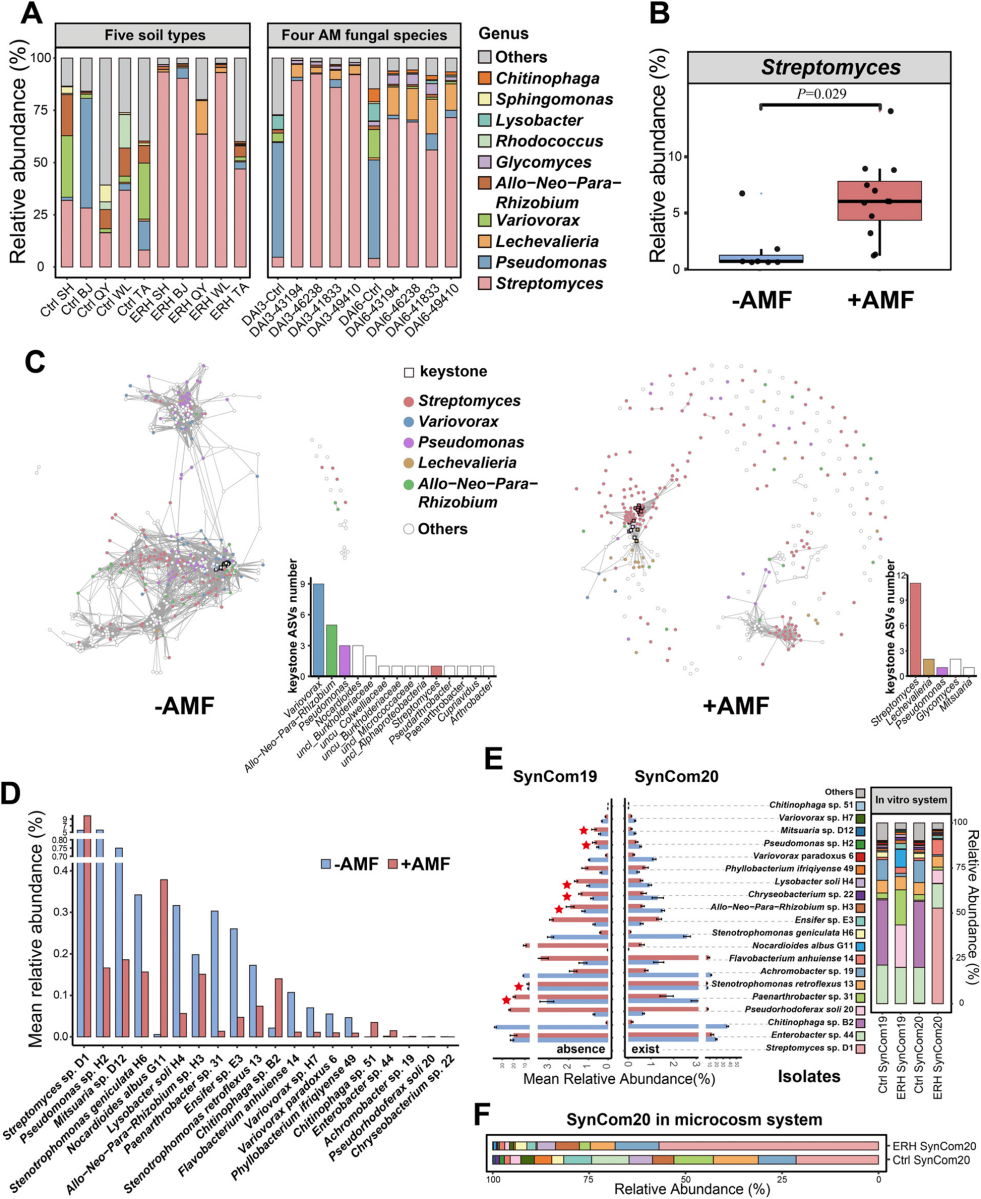
**A** Left: relative abundance (%) of the 10 most abundant bacteria genera associated with the extraradical hyphae (ERH) of *Rhizophagus irregularis* MUCL 43194 or developing in the absence of ERH (i.e., the control, Ctrl) 6 days after inoculation (DAI). The bacterial suspensions were extracted from soils of five long-term field experiments (soil SH: Shihezi 44°19′N, 86°00′E; soil BJ: Beijing 40°08′N, 116°10′E; soil QY: Qiyang 26°45′N, 111°52′E; soil WL: Wulumuqi 43°57′N, 87°46′E; soil TA: Taian 36°10′N, 117°09′E, SH, BJ, QY, WL, TA, respectively). Right: relative abundance (%) of the 10 most abundant genera associated with the ERH of four different AM fungi (i.e., *Rhizophagus irregularis* MUCL 43194, *Rhizophagus irregularis* MUCL 41833, *Rhizophagus clarus* MUCL 46238, and *Rhizophagus intraradices* MUCL 49410) or developing in the absence of ERH (i.e., the control, Ctrl), 3 or 6 DAI. Only the soil sampled in Beijing was considered. **B** Relative abundance (%) of *Streptomyces* species in the bulk soil without AM fungi (− AMF) or developing on the surface of hyphae/spores of AM fungi (+ AMF), based on data collected from six papers [23–28]. The host plants were carrot, maize, soybean, cotton, and alfalfa, and the AM fungal species belonged to *Glomus*, *Rhizophagus*, *Funneliformis*, and *Gigaspora*. The *t*-test was used for comparing the significant difference between the two treatments. **C** Bacterial communities co-occurrence networks visualizing significant correlations (*ρ* > 0.65, *P* < 0.01; indicated with gray lines) in controls (− AMF) and on the surface of ERH (+ AMF). Circles indicate bacteria amplicon sequence variants (ASVs). The gray edges represent strong and significant correlation between two nodes. Keystone ASVs (black-bordered square nodes) were identified separately for the − AMF and + AMF and defined as those nodes within the top 5% of node degree (number of edges correlations to a node) values of each network. Keystone ASVs are represented with black-bordered squares. ASVs are colored by their genus classification. The number of keystone ASVs in different genera in two bar chart alongside of co-occurrence networks. **D** Mean relative abundance (%) of ASVs (in five soil types and four fungal species) that match the 20 bacterial isolates from the soil BJ inoculated in the control (− AMF) or ERH (+ AMF) treatments. **E** Relative abundance (%) of bacteria in two SynComs associated (+ AMF) or not (− AMF) to the ERH of *Rhizophagus irregularis* MUCL43194 (SynCom19: 19 bacterial isolates without *Streptomyces* sp. D1; SynCom20: 19 bacterial isolates plus *Streptomyces* sp. D1). Error bars represent the standard error of five independent replicates. ERH-enriched bacterial isolates in the absence of *Streptomyces* sp. D1, and depleted by ERH in the presence of *Streptomyces* sp. D1, are symbolized by stars. **F** Relative abundance (%) of the 20 isolates associated with the ERH of *Rhizophagus irregularis* MUCL 43194 or developing in the absence of ERH as a SynComs (SynCom20) in the pot system

Bacterial suspensions extracted from the soils (bulk soil) of five long-term field experiments (soil SH, BJ, QY, WL, and TA) across China were spread on the surface of the hyphal compartment (HC) covered by extraradical hyphae (ERH) of *Rhizophagus irregularis* MUCL 43194 (i.e., ERH treatments) or free of hyphae (i.e., control treatments). Community composition was assessed by 16S rRNA gene profiling, 6 days after inoculation (DAI). Irrespective of soil origin, bacterial communities in the ERH treatments were dominated by the genus *Streptomyces* (Fig. 1A). Averaged over the five soils, this genus accounted for 77.5% ± 6.0% of the bacterial community. In the control treatments, a more even community (with Shannon evenness of 0.49 significantly higher than the Shannon evenness of 0.21 associated with ERH treatments) composition was noticed with the genus *Streptomyces* accounting for only 24.3% ± 4.2% of the bacterial community (Fig. 1A). The same approach was conducted comparing the bacterial community composition of ERH of four AM fungi (i.e., *R. irregularis* MUCL 43194, *R*. *irregularis* MUCL 41833, *Rhizophagus clarus* MUCL 46238, and *R*. *intraradices* MUCL 49410) and of the control treatments at 3 and 6 DAI. The soil BJ was considered. Regardless of DAI and AM fungus, the ERH treatments were dominated by the genus *Streptomyces*, while control treatments were dominated by the genus *Pseudomonas* (Fig. 1A). Averaged over the four AM fungal treatments, the genus *Streptomyces* accounted for 89.9% ± 1.1% and 67.0% ± 2.7% of the bacterial community at 3 and 6 DAI, respectively, while in the control treatments, it accounted for 4.7% ± 1.5% and 4.1% ± 0.7% of the bacterial community at 3 and 6 DAI, respectively.

We then collected data from six publications [23–28] to explore whether the preference for the genus *Streptomyces* is a widespread phenomenon. We compared the relative abundance of this genus in bulk and hyphosphere soil. The results showed that the relative abundance of the genus *Streptomyces* increased in the presence of AM fungi (Fig. 1B) suggesting a preponderance of AM fungi to recruit bacteria belonging to this genus.

Two bacterial community co-occurrence network analyses were further constructed in the presence versus the absence of ERH. In the presence of ERH, 17 keystone amplicon sequence variants (ASVs) were identified among which 11 belonged to the genus *Streptomyces* (Fig. 1C). The keystone ASVs of this genus showed more than 48 degrees of co-occurrence network, suggesting that they might influence the presence and distribution of other ASVs in the network. In the absence of ERH, 31 keystone ASVs were identified that mainly belonged to the genera *Variovorax*, *Allo-Neo-Para-Rhizobium*, *Pseudomonas*, and *Nocardioides*. Ten other genera, also including *Streptomyces*, were represented by only one keystone ASV (Fig. 1C).

To study the mechanisms involved in AM fungi and bacteria interaction, 62 bacteria were isolated from the ERH surface of *R*. *irregularis* MUCL 43194 (from the soil BJ), and 20 were selected by clustering at 97% similarity in 16S rRNA gene sequences to eliminate redundancy. The relative abundance of the 20 isolates on the ERH surface ranged from < 0.01 to 10.01% (Fig. 1D and Table S1). Interestingly, the mean relative abundance of ASVs that matched *Streptomyces* sp. D1 increased (from 5.62 to 10.01%) between the control treatments and the ERH treatments (Fig. 1D).

Finally, two SynComs were constructed, composed of 20 bacterial isolates (see for composition Fig. 1E), thus including (SynCom20) or not (SynCom19) *Streptomyces* sp. D1. The two SynComs were spread on the surface of the hyphal compartment (HC) covered by ERH of *R. irregularis* MUCL 43194 or free of hyphae. In the absence of *Streptomyces* sp. D1 (i.e., SynCom19), 12 isolates were enriched at the surface of the ERH, while in the presence of this bacterium (i.e., SynCom20), only 6 isolates were enriched (Fig. 1E). Due to the presence of *Streptomyces* sp. D1, 7 out of the 12 isolates that were enriched by ERH in SynCom19 were subsequently disfavored by ERH treatment, as indicated by a lack of bacterial growth (Fig. 1E, labelled with star). The SynCom20 was inoculated in the HC of the pot microcosm. In the presence of ERH, the relative abundance of *Streptomyces* sp. D1 was above 50%, while in the absence of ERH it was less than 25% (Fig. 1F). The *Streptomyces* sp. D1 was the most abundant in the ERH of the soil environment (Fig. 1F).

Collectively, the above results clearly showed that the genus *Streptomyces* was a keystone taxon in the early stage of interaction in the hyphosphere bacterial community of AM fungi under in vitro culture conditions.

### *Streptomyces* sp. D1 showed preference for trehalose as C source

**Fig. 2:**
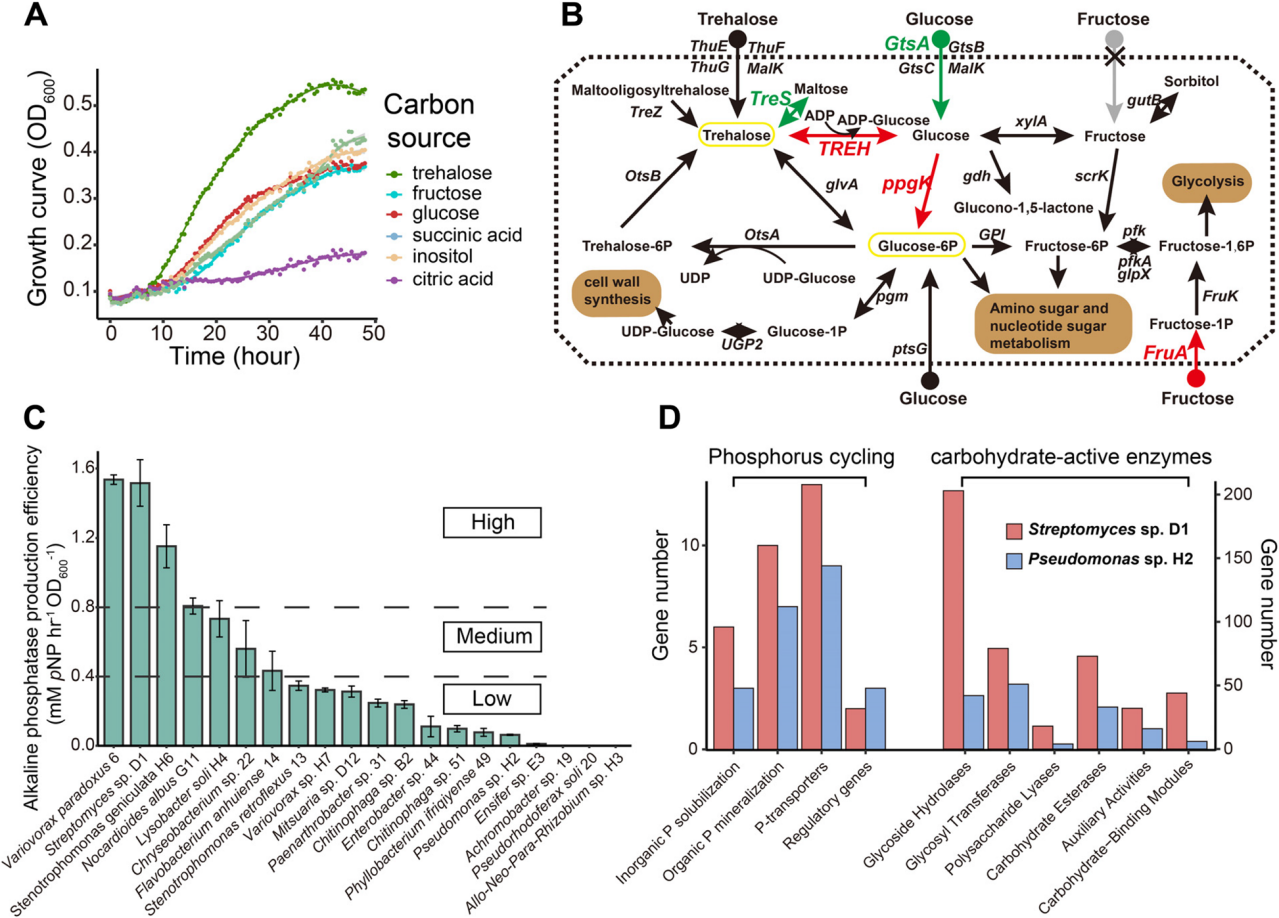
**A** Growth curves of *Streptomyces* sp. D1 in M9 minimal salts medium with trehalose, fructose, glucose, succinic acid, inositol, citric acid as carbohydrate source. **B** Genome map of *Streptomyces* sp. D1 for three major C sources (fructose, glucose, and trehalose) exuded by AM fungal hyphae and metabolic pathway colored with transcriptomic genes expression data. The genes with a significant (*P* < 0.05) differential expression of |log2FC|> 1 are indicated with an arrow (pathway) or gene name in green (down-regulated in contact with the extraradical hyphae (ERH)), red (up-regulated in contact with the ERH). Gray arrow represents the absence of genes identified in the pathway. **C** Alkaline phosphatase production efficiency of 20 isolates of bacteria isolated from the ERH of *Rhizophagus irregularis* MUCL 43194 inoculated with a bacterial suspension of the soil BJ. Error bars represent the standard error of five independent replicates. **D** Numbers of genes involved in P cycling and carbohydrate-active enzymes (CAZy) genes identified in genome of *Streptomyces* sp. D1 and *Pseudomonas* sp. H2

The carbon (C) assimilation profile of the 20 bacterial isolates was determined using six kinds of C sources (i.e., fructose, glucose, trehalose, inositol, citric acid, and succinic acid), which are among the major compounds found in the exudates of AM fungal hyphae [29–31]. Most bacteria were able to grow in the presence of at least one kind of C source and show preference for glucose, succinic acid, or citric acid (Figure S1). *Streptomyces* sp. D1 could use all six kinds of C sources, with a preference for trehalose (Fig. 2A). The whole-genome sequencing of *Streptomyces* sp. D1 showed the presence of genes encoding the proteins involved in trehalose transport (*ThuE*, *ThuF*, *ThuG*, and *MalK*) and metabolism (*OtsB*, *TREH*, *TreZ*, *TreS*, and *glvA*) (Fig. 2B and Table S2). The transcriptional sequence results showed that in the presence of ERH of *R. irregularis* MUCL 43194, the expression of genes from trehalose to glucose-6-P of *Streptomyces* sp. D1, including α-trehalase gene *TREH* (converting trehalose to glucose) and polyphosphate glucokinase gene *ppgK* (converting glucose to glucose-6P), was significantly increased (Fig. 2B).

### *Streptomyces* sp. D1 had a strong ability to mineralize organic P in the hyphosphere

The alkaline phosphatase (AP) activity of the 20 bacterial isolates was measured under C (10-mM glucose) and P (KH_2_PO_4_, 50 mM) limited condition via the release of *para*-nitrophenol (*p*NP) from *para*-nitrophenol phosphate (*p*NPP). The bacteria were arbitrarily separated in three groups (i.e., high > 0.8, 0.4 < medium < 0.8, and low < 0.4-mM *p*NP hr^−1^ OD_600_^−1^) according to their efficacy to produce AP (Fig. 2C), with bacteria having a high AP being significantly different from those with low AP (Tukey’s HSD, *P* < 0.05). *Streptomyces* sp. D1 ranked in the highest AP production efficiency, while *Pseudomonas* sp. H2 had nearly the lowest AP production efficiency (Fig. 2C).

From the genome analysis, *Streptomyces* sp. D1 had abundant P metabolism genes, including 10 genes related to organic P mineralization, 9 genes related to inorganic P solubilization, 14 genes related to P transporters, and 2 genes related to P metabolism regulation (Fig. 2D). *Streptomyces* sp. D1 also had genes (e.g., GH6, PL1, PL3, and PL9) involved in the degradation of plant cell wall polysaccharides (Fig. 2D), which are absent in the genome of *R*. *irregularis* (Additional file 2). Overall, and with the exception of the regulatory genes, the number of genes involved in P metabolism (inorganic P solubilization, organic P mineralization, and P transporters), cellulose or hemicellulose degradation (glycoside hydrolases, GHs; glycosyl transferases, GTs; polysaccharide lyases, PLs; carbohydrate esterases, CEs; auxiliary activities, AAs; carbohydrate-binding modules, CBMs) was higher in the genome of *Streptomyces* sp. D1 compared to the genome of *Pseudomonas* sp. H2 (Fig. 2D, Table S3, Table S4, and Additional file 2).

**Fig. 3:**
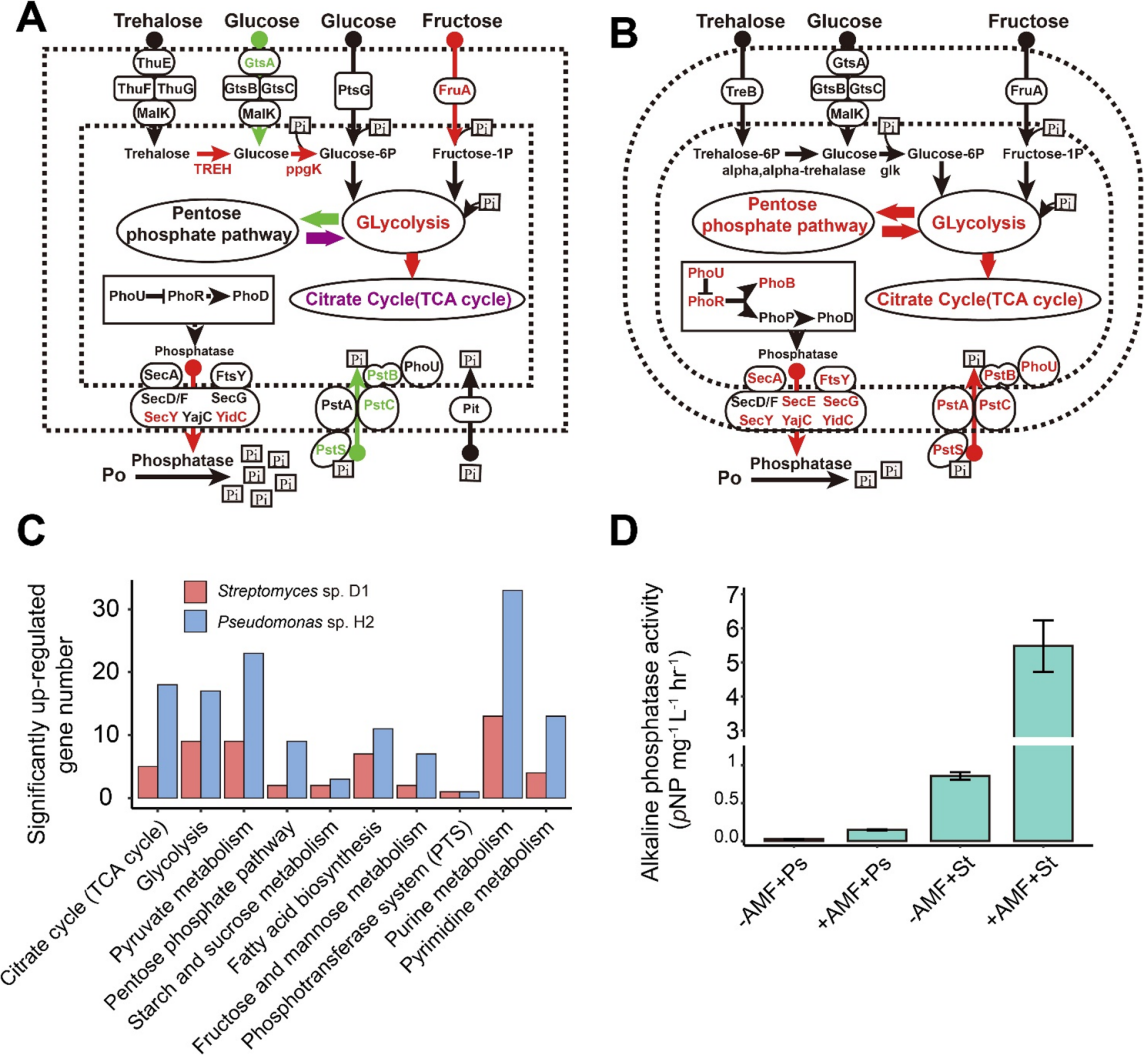
Major carbohydrate and phosphate metabolic pathways mapped with transcriptomic data in **A** *Streptomyces* sp. D1 and **B** *Pseudomonas* sp. H2. The reconstructed metabolic pathways were based on KEGG gene annotations and their relative differential gene expression profiles of triplicates (each replicate was a mix of 5 plates, 15 plates for every treatment). The genes with a significant (*P* < 0.05) differential expression of |log2FC|> 1 are indicated with an arrow (pathway) and gene name in green (down-regulated in contact with the extraradical hyphae (ERH)), red (up-regulated in contact with the ERH), purple (some genes down-regulated, and some genes up-regulated in contact with the ERH). **C** Numbers of up-regulated genes involved in carbon and phosphorus consumption in *Streptomyces* sp. D1 and *Pseudomonas* sp. H2 associated with ERH compared to controls. **D** Alkaline phosphatase activity of *Streptomyces* sp. D1 and *Pseudomonas* sp. H2 in contact (+ AMF) or not (− AMF) with the ERH of *Rhizophagus irregularis* MUCL43194 (*n* = 3, each replicate was a mix of five plates). Ps, *Pseudomonas* sp. H2; St, *Streptomyces* sp. D1

*Streptomyces* sp. D1 and *Pseudomonas* sp. H2 were the dominant organisms in the bacterial community in the presence and absence of AM fungal hyphae, respectively, in soil BJ. Therefore, to determine the influence of AM fungi on the organic P mobilization ability of *Streptomyces* sp. D1 and *Pseudomonas* sp. H2, a comparative mRNA transcriptomic analysis was performed on both bacteria in the presence or absence of ERH of *R*. *irregularis* MUCL 43194. Overall, 768 and 1159 genes were significantly differentially expressed (*P* < 0.05) between these two conditions in *Streptomyces* sp. D1 and *Pseudomonas* sp. H2, respectively. Numerous genes associated with energy production were up-regulated in the glycolysis and citrate cycles in both microorganisms, indicating that a huge flow of C was transferred from the AM fungus to the bacteria (Figure S2A, B). The up-regulation of fructose phosphotransferase gene *fruA* and alpha-trehalase gene *TREH* (Fig. 3A) suggested that the hyphae released fructose and trehalose were taken up by *Streptomyces* sp. D1 resulting in genes up-regulated in the glycolysis and citrate cycle pathways. Furthermore, the inorganic P transporters, including *pstS*, *pstC*, and *pstB*, were down-regulated in *Streptomyces* sp. D1 (Fig. 3A). Most of the genes involved in glycolysis, citrate cycle, and pentose phosphate pathway were significantly increased in *Pseudomonas* sp. H2 (Fig. 3B and Figure S2B), as well as its inorganic P transporter genes (*pstS*, *pstC*, *pstA*, and *pstB*) (Fig. 3B). The inorganic P solubilization gene *gcd*/*gdh* and organic P mineralization gene *phoD* were not significantly changed in both strains grown in contact with the ERH (Fig. 3A, B; Figure S2A, B). Much more genes of C (glycolysis, citrate cycle, and pentose phosphate) and P (purine metabolism and pyrimidine metabolism) consumption pathways were up-regulated in *Pseudomonas* sp. H2 than in *Streptomyces* sp. D1 (Fig. 3C). The Sec secretion pathway (including genes: *secA*, the ATPase motor; *ftsY*, membrane receptor; *secYEG*, transmembrane SecYEG channel; *secDF*, *yajC*, and *yidC*, auxiliary component enhance translocation efficiency) is ubiquitous in all domains of bacteria that can secrete enzymes (e.g., AP). The genes of *secY* and *yidC* were significantly up-regulated in both bacteria (Fig. 3A, B). This was consistent with the stronger activity of AP observed in the two bacteria in contact with ERH compared to the control. *Streptomyces* sp. D1 showed much stronger AP (more than 37 times) than *Pseudomonas* sp. H2 in contact with ERH (Fig. 3D). Furthermore, 17 out of 89 cell motility genes were up-regulated in *Pseudomonas* sp. H2 indicating that motility was activated in the presence of ERH exudates (Table S5 and Additional file 3).

### *Streptomyces* sp. D1 stimulated organic P utilization by AM fungi and gene expression of C-P exchange in plants

**Fig. 4:**
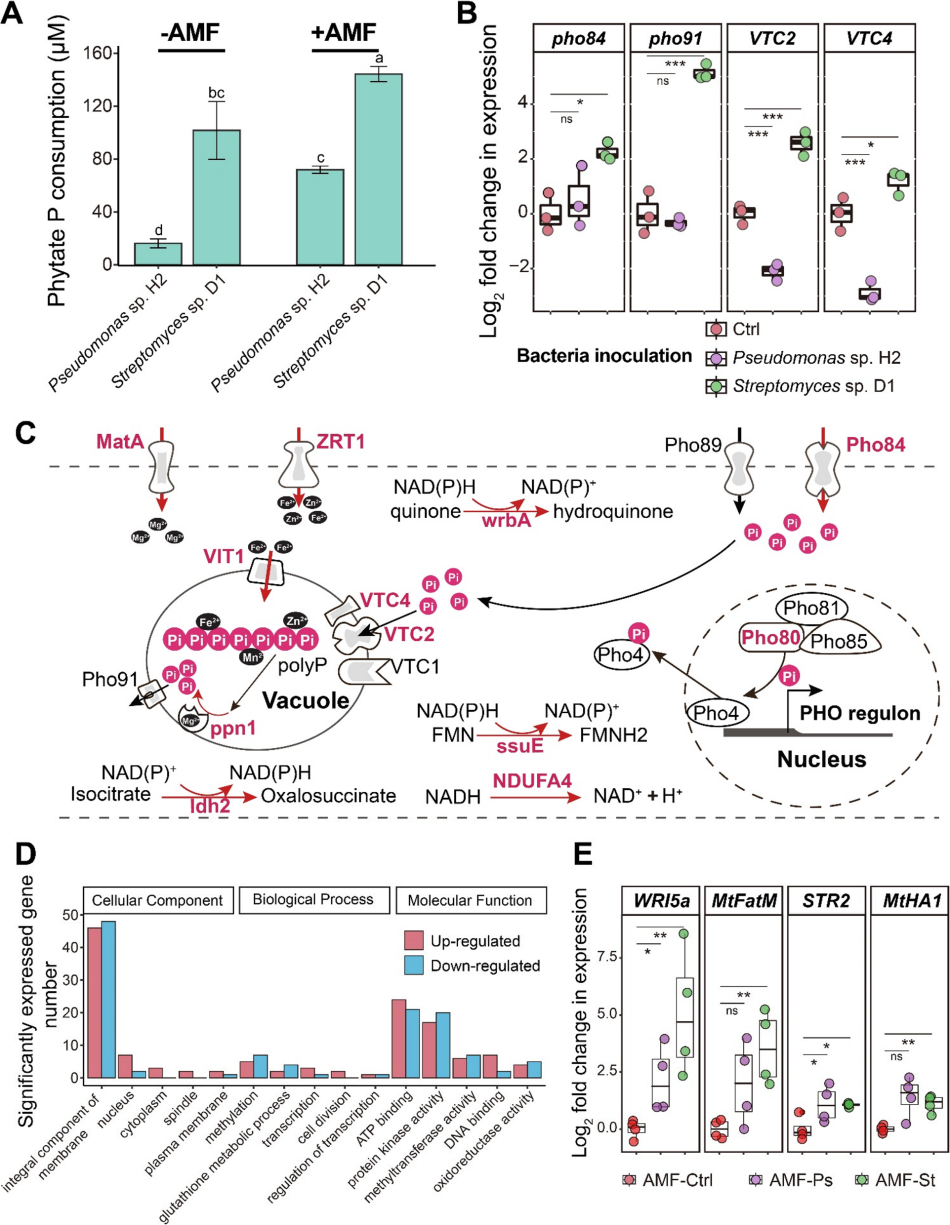
**A** Phytate-P consumption of *Streptomyces* sp. D1 and *Pseudomonas* sp. H2 in the absence (− AMF) or presence (+ AMF) of the ERH of *Rhizophagus irregularis* MUCL 43194. The least significant difference (LSD) test was used to identify the differences in P consumption at 0.05 significance level. Error bars represent the standard error of three independent replicates. **B** Gene expression of *Pho84*, *Pho91*, *VTC2*, and *VTC4* in the ERH of *R. irregularis* MUCL 43194 in the absence of bacteria (Ctrl) or inoculated with *Streptomyces* sp. D1 or *Pseudomonas* sp. H2 (*n* = 3 biologically independent samples, ***P < 0.001, **P < 0.01, *P < 0.05, LSD). **C** Summary of the significantly up-regulated genes in *R. irregularis* MUCL 43194 involved in phosphate, polyphosphate, and energy turnover inoculated with *Streptomyces* sp. D1 as compared to the fungus grown in the absence of the bacteria. The genes with a significant (*P* < 0.05) differential expression of log2FC > 1 are indicated with gene name in red (up-regulated in contact with *Streptomyces* sp. D1). **D** Differentially expressed gene number (*P* < 0.05, |log2FC|> 1) in cellular components, biological processes, and molecular functions of the ERH of *R. irregularis* MUCL 43194 inoculated with *Streptomyces* sp. D1 as compared to the fungus grown in the absence of the bacterium. **E** Gene expression of *WRI5a*, *MtFatM*, *STR2*, and *MtHA1* in *Medicago truncatula* associated with the ERH of *R. irregularis* MUCL 43194 in the absence of bacteria (Ctrl) or inoculated with *Streptomyces* sp. D1 or *Pseudomonas* sp. H2 (*n* = 4 biologically independent samples, ***P < 0.001, **P < 0.01, *P < 0.05, LSD)

The phytate-P consumption of *Streptomyces* sp. D1 and *Pseudomonas* sp. H2 was significantly enhanced when the bacteria were grown in contact with the ERH of *R. irregularis* MUCL 43194 versus their growth in the absence of ERH (Fig. 4A). Moreover, in the presence of the ERH, the phytate consumption of *Streptomyces* sp. D1 was nearly doubled as compared to *Pseudomonas* sp. H2, and in the absence of ERH, it was six-folds greater (Fig. 4A). Gene expression of Pi-transporter (*Pho84*), vacuolar phosphate transporter (*Pho91*), and vacuolar transporter chaperone (*VTC2* and *VTC4*) was significantly greater in the ERH inoculated with *Streptomyces* sp. D1 as compared to the control (i.e., the ERH without the bacteria — Fig. 4B). Reversely, in the presence of *Pseudomonas* sp. H2, a significant down-regulation was noticed for *VTC2* and *VTC4* compared to the control. No differences in gene expression in the presence/absence of *Pseudomonas* sp. H2 were noticed for *Pho84* and *Pho91* compared to the control (Fig. 4B).

A comparative mRNA transcriptome was conducted on triplicate ERH samples (each replicate was a mix of 5 plates, 15 plates for each treatment) of *R. irregularis* MUCL 43194 associated or not to *Streptomyces* sp. D1. More than 1.6% of the AM fungal gene repertoire (434 out of 26143 genes) was affected by the bacterium. Twenty genes, known to be necessary for phosphate and polyphosphate transport and metabolism in *R. irregularis*, were scrutinized. A general increase of expression of genes involved in phosphate limitation regulated gene (*pho80*) phosphate transport (*pho84*) and polyphosphate synthesis/decomposition (*VTC2*, *VTC4*, and *PPN*1) was noticed in the presence of *Streptomyces* sp. D1 (Fig. 4C). In addition, the expression of polyphosphate-related ion transporter genes including *ZRT1* (zinc-regulated transporter 1), *MatA* (magnesium transporting ATPase), and *VIT1* (vacuolar iron transporter 1) was up-regulated in the presence of the bacterium (Fig. 4C). A general increase in NAD(P)H synthesis and oxidoreduction was also observed (Fig. 4C).

The differentially expressed genes were divided into Gene Ontology (GO) functional categories. This included 112 genes associated to cellular components, 49 genes to biological processes, and 136 genes to molecular functions (Fig. 4D). The genes most up- or down-regulated belong to the integral component of membrane, the ATP binding, and protein kinase activity. This included 46 genes up-regulated and 48 genes down-regulated (integral component of membrane), 24 genes up-regulated and 21 genes down-regulated (ATP binding), and 17 genes up-regulated and 20 genes down-regulated (protein kinase activity) in the ERH of the AM fungus inoculated with *Streptomyces* sp. D1 as compared to the fungus grown in the absence of the bacterium, respectively (Fig. 4D).

The effect of bacteria on AM fungi could be extended to the plant. After inoculation of *Streptomyces* sp. D1 in the hyphosphere, a significant increase in the expression of the ATPase gene *MtHA1* that is essential for P transport from the symbiotic interface to the plant was observed. Similarly, the expression of genes related to fatty acid synthesis (*WRI5a* and *MtFatM*) and transport (*STR2*) from plants to symbiotic interface was significantly increased (Fig. 4E). The inoculation of *Pseudomonas* sp. H2 in the hyphosphere also significantly increased the expression of *WRI5a* and *STR2* in the plant, while no differences were noticed for the expression of genes *MtFatM* and *MtHA1* (Fig. 4E).

### *Streptomyces* sp. D1 impacted the bacterial community in the hyphosphere

**Fig. 5:**
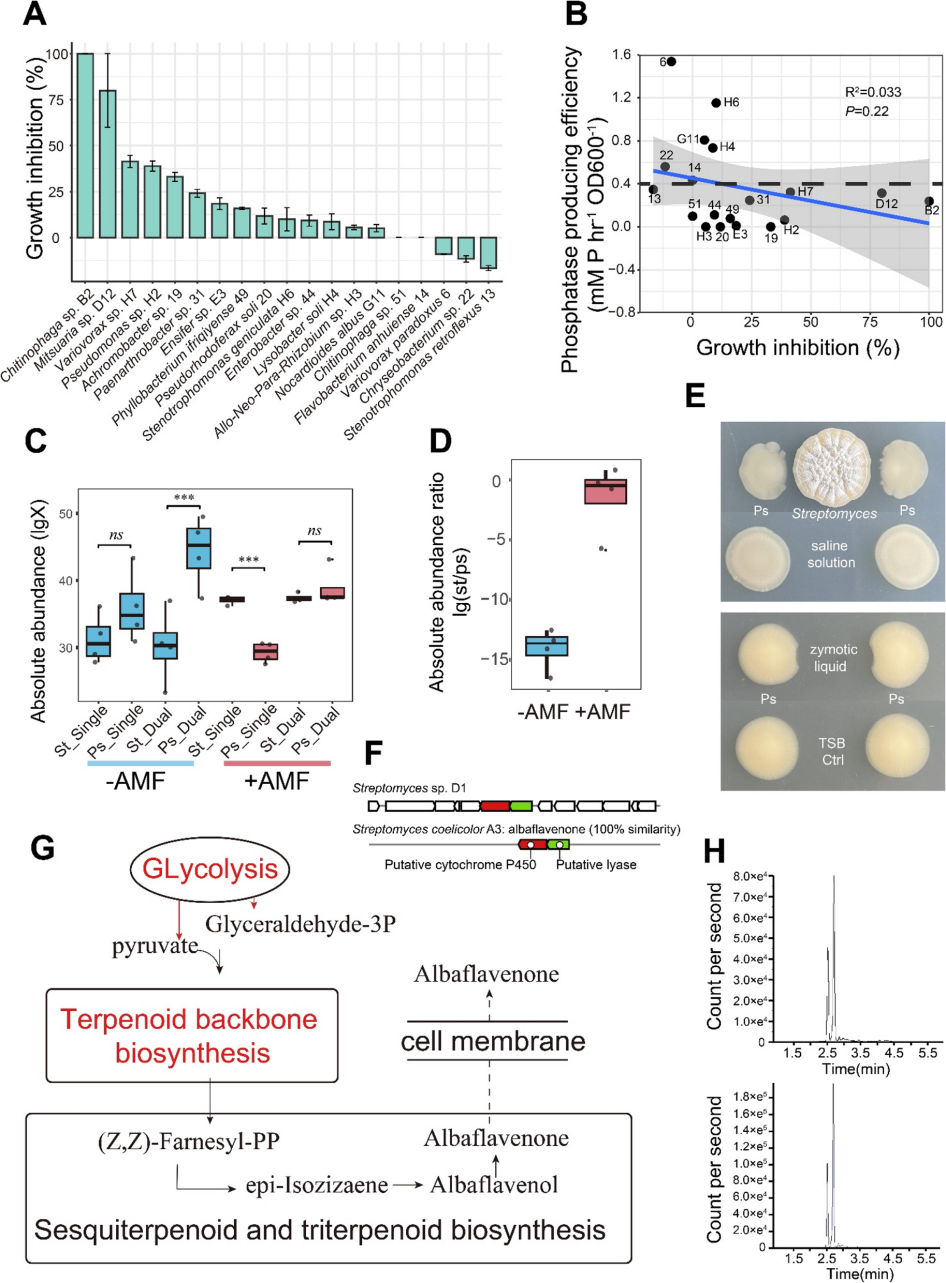
**A** Growth inhibition of *Streptomyces* sp. D1 exudates on 19 bacteria isolated from the surface of the ERH cultured in contact with the bacterial suspension of soil BJ. **B** Correlation of growth inhibition and phosphatase producing efficiency of 19 bacteria isolated from the surface of the extraradical hyphae (ERH) of *Rhizophagus irregularis* MUCL 43194. **C** Absolute abundance of *Streptomyces* sp. D1 and *Pseudomonas* sp. H2 growing singly or dually, in contact (+ AMF) or not (− AMF) with the ERH of *R. irregularis* MUCL 43194 (*n* = 4 biologically independent samples, ***P < 0.001, **P < 0.01, *P < 0.05, LSD). **D** Ratio of the absolute abundance of *Streptomyces* sp. D1 and *Pseudomonas* sp. H2 in the absence (left bar) or presence (right bar) of ERH in dual culture conditions. **E** Impact of *Streptomyces* sp. D1 (top) or its zymotic liquid (down) on *Pseudomonas* sp. H2 (Ps) on solid medium (0.5% peptone, 0.3% yeast extract, 0.5% NaCl, 0.8% agar). Ctrl is the control with 0.9% NaCl (w:v) solution or TSB medium. **F** Albaflavenone biosynthesis gene cluster detected by antiSMASH6 in genome of *Streptomyces* sp. D1. **G** Reconstruction of the *Streptomyces* sp. D1 terpenoid backbone biosynthesis and sesquiterpenoid and triterpenoid biosynthesis pathways mapped with transcriptomic data. Reconstructed metabolic pathway of the *Streptomyces* sp. D1 based on KEGG gene annotations and its relative differential gene expression profiles of triplicates (each replicate was mixed by 5 plates, 15 plates for every treatment). The pathways with genes significant (*P* < 0.05) differential expression of log2FC > 1 are indicated with pathway name in red (up-regulated in contact with the ERH). **H** UPLC-MSMS analysis of metabolites extracted from the 1/2 TSB medium cultured S*treptomyces* sp. D1. UPLC-MSMS chromatograms of albaflavenone standard (top) and from the growth medium (TSB, tryptone soy broth) of *Streptomyces* sp. D1 (down)

Exudates of *Streptomyces* sp. D1 were tested for growth inhibition on 19 bacteria from the surface of ERH grown with soil BJ bacterial suspension. Fourteen out of 19 isolates, including *Pseudomonas* sp. H2, were inhibited by the exudates (Fig. 5A). Notably, six isolates with higher AP production efficiency (> 0.4 mM *p*NP h^−1^ OD_600_^−1^) were not impacted or minimally inhibited (Fig. 5B). A negative correlation was found between *Streptomyces* sp. D1 and *Pseudomonas* sp. H2 in soil BJ. qPCR showed a significant decrease in the absolute abundance ratio of *Streptomyces* sp. D1 and *Pseudomonas* sp. H2 in the absence of ERH. *Streptomyces* sp. D1 had greater colony development in ERH treatment in single or dual culture than in the control treatment (Fig. 5C, D). Both the culture cells and exudates of *Streptomyces* sp. D1 were able to inhibit the normal growth of *Pseudomonas* sp. H2 (Fig. 5E).

The *Streptomyces* sp. D1 harbored the entire albaflavenone (bactericidal antibiotic) synthesis pathway genes (Fig. 5F, G and Table S6). Consistently, a general increase of genes involved in terpenoid backbone biosynthesis in *Streptomyces* sp. D1 was observed, which were necessary for synthesis of albaflavenone precursor substances (Fig. 5G and Figure S3). Additionally, albaflavenone (C_15_H_22_O) at the concentration of 0.93 µg L^−1^ (*R*^2^ = 0.998) was identified by UPLC-MSMS against the standard (CAS: 157078–47-2) on the 1/2 TSB medium cultured *Streptomyces* sp. D1 (Fig. 5H).

## Discussion

The roots of most soil-grown plants are colonized by AM fungi with their ERH inhabited by structured communities of bacteria that provide direct benefits to their fungal host (e.g., solubilization of organic P) in exchange for C resources [16]. As a result, the interaction between AM fungi and bacteria along the soil-hyphae-root continuum and microbe-microbe interactions on the hyphal surface has gradually received increased attention in recent years. Besides the endobacterium living inside an AM fungus which has been demonstrated to have a strong impact on both the fungal and plant associates [32, 33], the hyphosphere bacteria have been shown to have a significant impact on the nutrient acquisition and fitness of AM fungi [19, 20, 34–37]. The study of the hyphosphere microbiome (hyphobiome) of AM fungi requires to consider not only the interactions between AM fungi and their associated bacteria but also those between different species or functional groups of bacteria living at the hyphal surface [38, 39]. Here, we showed that 4 AM fungal strains are capable to preferentially recruit a specific bacterial taxon (i.e., *Streptomyces* sp. D1), from a community of in vitro culturable bacteria, in their hyphosphere, improving their nutrition and rewarding it with hyphal exudates. This selection further benefits the association between AM fungi and the plant by increasing the expression of genes involved in C-P exchange at the symbiotic interface. Moreover, we demonstrated that *Streptomyces* sp. D1 is a keystone taxon that plays an important role in shaping the bacterial community structure in the early stage of interaction at the surface of AM fungal hyphae, partly inhibiting the growth of bacteria with weak P-mineralizing ability, and noted that its removal can lead to significant changes in microbiome composition and functioning.

### AM fungi preferentially recruits *Streptomyces* sp. from a community of in vitro culturable bacteria

Our study demonstrated that the ERH of AM fungi harbors a highly diverse assemblage of bacteria, further supporting evidence that hyphae release exudates that provide an energy-rich microhabitat shaping bacterial community composition in the same way as root exudates [29, 30]. Thus, AM fungal hyphae can be an important source of nutrients for a wide range of bacteria. The leachates of five soils inoculated in vitro on the ERH of *R. irregularis* MUCL 43194 or a single leachate of soil inoculated on the ERH of four different AM fungi showed a predominance of the genus *Streptomyces* over the other bacterial taxa. This was supported by data collected in other studies [23–28] conducted in vitro and in soil showing a clear enrichment of the genus *Streptomyces* by the presence of AM fungi, which can be considered as “fungiphile”, as suggested by Warmink and van Elsas [40] for other bacteria closely associated with soil fungi. In the present study, we analyzed the bacterial community at the early stage of interaction with the hyphae of AM fungi and found that *Streptomyces* sp. was a bacterium that grew rapidly in response to the presence of AM fungi. These early bacterial communities play a decisive role during the “establishment phase”, setting the stage for later community assemblages. Understanding this nascent community is crucial to understand the selection pressures exerted by AM fungi. The dynamics of early establishment on the hyphal surface reflects AM fungal selection, before other environmental factors affect these interactions. Long-term interaction studies are needed to determine whether these bacteria will remain dominant in the hyphosphere bacterial community. It should be noted, however, that under soil conditions, studies have shown that Proteobacteria, Actinobacteria, and Myxococcota phyla are enriched by AM fungi. In addition, a considerable proportion of Actinobacteria have been attributed C by AM fungi or have been defined as core species [21, 39, 41, 42]. The differences noticed between our in vitro culture system and the studies conducted under soil conditions may be attributed to the following reasons: First, the bacterial community in soil is much larger than in in vitro that only comprise culturable bacteria. Second, the environmental conditions markedly differ between soil and in vitro systems. Indeed, the soil is a complex micro-niche composed of solid, liquid, and gas phases and may also comprise a number of uncontrolled or less controlled factors (e.g., unwanted microbial contaminants). In its defense, the in vitro culture system is the only that makes it possible to study the bacteria-bacteria and bacteria-AM fungi interactions in a very rigorous way (i.e., under strictly controlled conditions). Thirdly, the bacterial community investigated in the present study is at the very early stage of interaction on the hyphal surface. It is therefore suggested that early growers may be more likely to dominate the bacterial community.

The preponderance of *Streptomyces* sp. D1 in our study could be related partly to its ability to assimilate fructose, glucose, trehalose, inositol, citric acid, and succinic acid, which are among the major compounds found in the exudates of AM fungal hyphae [29–31]. Most of the other bacteria tested (e.g., *Roseateles*, *Pseudomonas*, *Allo-Neo-Para-Rhizobium*, see Figure S1) were able to grow in the presence of at least one C source and show preference for glucose, succinic acid, or citric acid (Figure S1). *Streptomyces* sp. D1 showed preference for trehalose, suggesting that this compound was favorably used by this bacterium in the hyphosphere. This was supported by the trehalose transporter clusters found in the genome of *Streptomyces* sp. D1. The genes for synthesis of trehalose, trehalose 6-phosphate synthase (*otsA* and *TPS*), are present in the genome of *R*. *irregularis* MUCL 43194. However, no significant change was observed at the transcriptomic level. Previous studies have detected glucose, fructose, and trehalose in the secretion of hyphae. In particular, trehalose is highly abundant within hyphae and possesses the potential to be released extracellularly.

The preponderance of *Streptomyces* sp. D1 on the hyphal surface may also be related to the nutritive advantage that the AM fungi may derive from this association. Zhang et al. [16, 37] have already demonstrated that the PSB *Rahnella aquatilis* was able to mineralize organic P (i.e., phytate) into inorganic P, stimulating the processes involved in P uptake by *R*. *irregularis*. In exchange, the AM fungus rewarded the bacterium with fructose, which could be considered as a C source but also as a signal molecule triggering bacteria-mediated organic P mineralization processes. On the basis of the growth curve and the AP production efficiency of the bacteria, it was observed that at a glucose concentration of 10 mM, the bacterial culture remained stable for 48 h, indicating glucose depletion. In addition, *Streptomyces* sp. D1 showed the highest efficiency of AP production compared to the other bacteria. This implied that if the AM fungus was able to invest the same amount of C in *Streptomyces* sp. D1, it would obtain the greatest reward in terms of P mobilization from this bacterium. In addition, as a consequence of the organic P mobilization by *Streptomyces*, the AM fungal genes involved in P uptake and polyP synthesis were up-regulated. Collectively, these results suggested that the AM fungi receive direct benefits from *Streptomyces* sp. D1 by increasing P availability.

### *Streptomyces* sp. D1 is a keystone taxon shaping the bacterial community structure at the surface of AM fungal hyphae

We constructed microbial co-occurrence networks based on soil samples to investigate the interactions among bacteria. *Streptomyces* species were found to be keystones taxa in the network and played a major role in constructing the microbial network. Synthetic microbial communities were also designed to verify the results based on co-occurrence network. *Streptomyces* sp. D1 strongly altered the bacterial community and inhibited the growth of certain bacterial isolates, especially those with low AP production efficiency. For instance, the relative abundance of *Paenarthrobacter* sp. 31 decreased from 19.4% in the ERH-SynCom without *Streptomyces* sp. D1 to 1.7% in the presence of *Streptomyces* sp. D1.

Besides the C for P exchange demonstrated between the AM fungi and *Streptomyces* sp. D1 (see above), the AM fungi could indirectly reward *Streptomyces* sp. D1 with C for its ability to compete with other bacteria using inorganic P sources for their own needs when they utilize the hyphal exudates [19, 43], thereby competing with AM fungi for P availability [44, 45]. Although no significant negative correlation was noticed between the AP production efficiency of the bacterial isolates and the growth inhibition by the exudates of *Streptomyces* sp. D1 (*R*^2^ = 0.033, *P* = 0.22, Fig. 5B), all the isolates having an AP production efficiency above 0.4 mM *p*NP h^−1^ OD_600_^−1^ had their growth either stimulated, not impacted, or only slightly inhibited by the exudates of *Streptomyces* sp. D1. This suggested that *Streptomyces* sp. D1 may spare or at least not impact too heavily the bacteria with the highest organic P mobilization capability and thus those bacteria that were not truly competing with AM fungi for P.

Interestingly, in the interaction between *Streptomyces* sp. D1 and *Pseudomonas* sp. H2, the ratio of absolute abundance of both bacteria at the hyphal surface was in favor of *Streptomyces* sp. D1, while in the absence of hyphae, the contrary was noticed. *Pseudomonas* sp. H2 has a very low AP production efficiency, therefore relying on the P mineralized by other bacteria with strong AP production efficiency. Furthermore, this bacterium inhibited polyP synthesis and transport processes in the ERH of the AM fungus. The exudates of *Streptomyces* sp. D1 inhibited the growth of *Pseudomonas* sp. H2 by nearly 40%, therefore decreasing the competitive pressure of this bacterium with the AM fungus for P. Using the whole genome sequence of *Streptomyces* sp. D1, multitudinous secondary metabolism genes were identified, related to antibiotics, including types I, II, and III polyketide synthases, non-ribosomal peptide synthases, and terpene (Additional file 1 Table S6). Albaflavenone (C_15_H_22_O), a tricyclic sesquiterpene, was detected in the exudates of *Streptomyces* sp. D1 (Fig. 5F and Additional file 1 Table S6) and reported to have strong antibacterial efficiently. *Streptomyces* sp. D1 could thus assist the AM fungus which lack the ability to inhibit the weak P-mineralizing bacteria [46].

### AM fungi and *Streptomyces* sp. D1: a global perspective

**Fig. 6:**
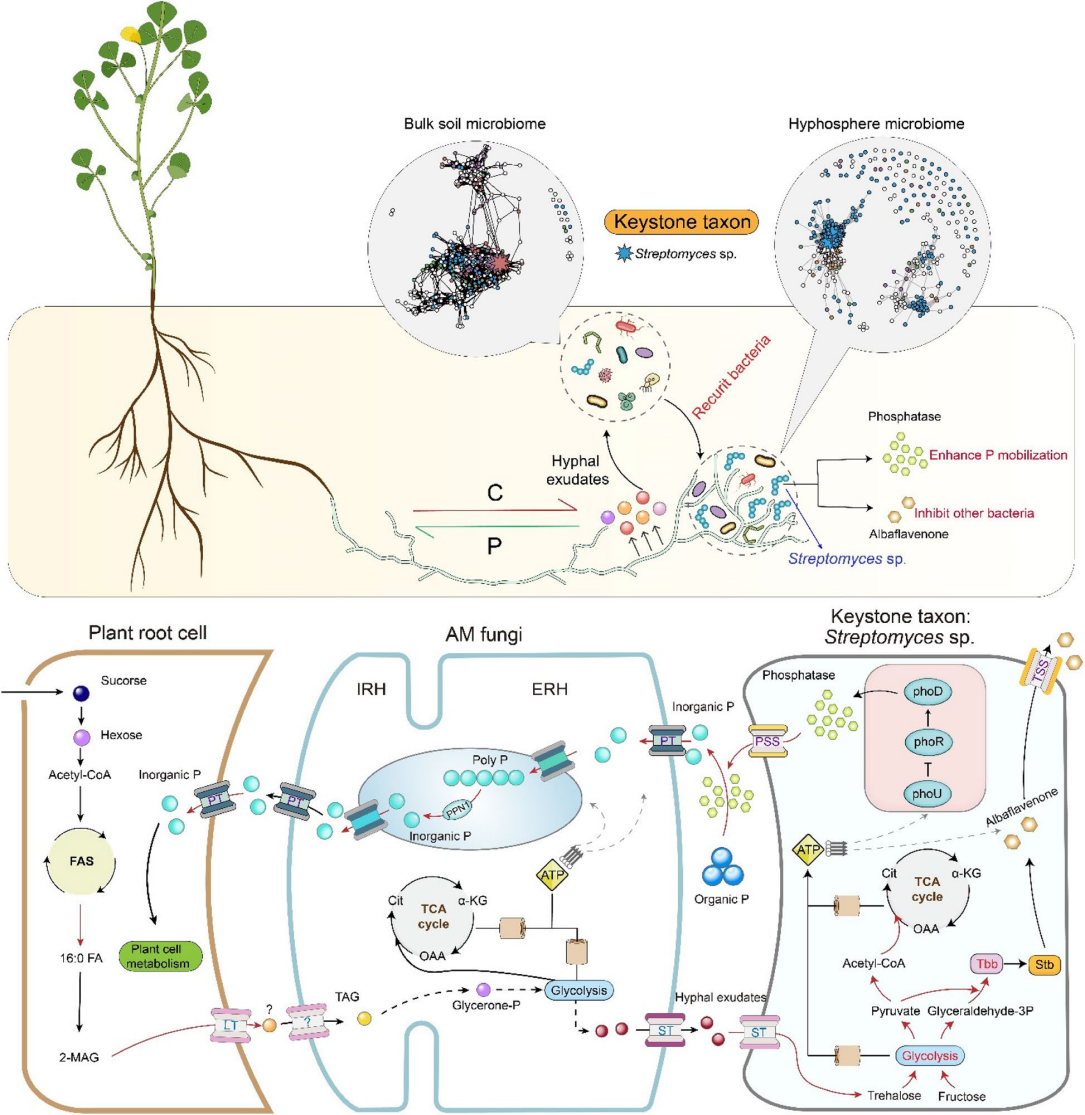
Schematic representation of the complex interactions between AM fungi and bacteria and bacteria-bacteria on the surface of AM fungal hyphae. The up-regulated genes or pathway is in red. **A** series of genes from *Streptomyces* sp. D1 to plant cell (phosphorus turnover) and genes from plant cell to *Streptomyces* sp. D1 (C turnover) were described. *Streptomyces* sp. D1 occupied the hyphosphere and used its albaflavenone antibiotic to regulate the bacterial community structure on the hyphal surface. IRH, intraradical hyphae; ERH, extraradical hyphae; PT, phosphate transporter; PSS, protein secretory system; TSS, toxin secretory system; LT, lipid transporter; ST, sugar transporter

Together, our results demonstrate the complex interactions between AM fungi and bacteria and bacterial-bacterial interactions on the surface of AM fungal hyphae. We showed that under the organic P condition, AM fungi are able to preferentially recruit from a community of in vitro culturable bacteria, species that improve their P nutrition and compete with weak P-mineralizing bacteria in the early stage (Fig. 6). Thus, a privileged interaction was established with *Streptomyces* sp. D1; the ERH of AM fungi release C compounds, which are acquired by *Streptomyces* sp. D1 in exchange for mineralized organic P. This keystone bacterium also impacted the bacterial community residing on the hyphal surface, primarily by inhibiting bacteria with low AP production efficiency, thus competing with AM fungi for P. Furthermore, we shed light on the role of hyphae in connecting plants to bacteria. Indeed, the benefits of bacteria to AM fungi can be transmitted to plants via the hyphae, which greatly enhances our understanding of a novel paradigm: AM fungi serving as a bridge to connect plants and bacterial communities.

## Methods

### Soil material and extraction of bacteria

Soils were sampled in five long-term field experiments (soil SH: Shihezi 44°19′N, 86°00′E; soil BJ: Beijing 40°08′N, 116°10′E; soil QY: Qiyang 26°45′N, 111°52′E; soil WL: Wulumuqi 43°57′N, 87°46′E; soil TA: Taian 36°10′N, 117°09′E), which are located west (SH and WL), east (BJ and TA), and south (QY) of China. In each location, five subsamples of circa 300 g of soil were taken with an auger at a depth of 0 to 20 cm, mixed together, then sieved to 2 mm, and finally homogenized to constitute the soils SH, WL, BJ, TA, and QY. Soils were transported to the laboratory in sterile plastic bags on ice and temporarily stored at 4°C until extraction of bacterial suspensions. The texture, N, P, K, and organic C contents of the soils are detailed in Table S7. Bacterial suspensions were extracted from the soils using Tween 80/tetrasodium yrophosphate (TTSP) and Nycodenz [47–50] (for details, see Methods in the Additional file 1).

### Arbuscular mycorrhizal fungi

Four AM fungal stains, i.e., *Rhizophagus irregularis* MUCL 43194, *Rhizophagus irregularis* MUCL 41833, *Rhizophagus clarus* MUCL 46238, and *Rhizophagus intraradices* MUCL 49410, were provided by the Glomeromycota in vitro collection (GINCO, Belgium). The fungi were maintained in vitro on Ri T-DNA transformed roots of carrot (*Daucus carota* L.) clone DC2 on the modified Strullu-Romand (MSR) medium [51] as described in Cranenbrouck et al. (2005) [52].

### In vitro culture system design

Bi-compartmented Petri plates (90 × 15 mm) were used to grow the carrot roots and AM fungi as detailed in St Arnaud et al. (1996) [53] (Figure S4A, B). Briefly, in one compartment (i.e., the root compartment — RC), the root was associated to the AM fungus, while in the other compartment (i.e., the hyphal compartment — HC), only the extraradical hyphae (ERH) of the AM fungus were allowed to growth. Twenty-five milliliters of sterilized (121°C for 15 min) MSR medium solidified with 3 g L^−1^ Phytagel (Gelzan™ CM, G3251, CP Kelco, USA) was added in the RC. In the HC, 20 mL of the modified MSR medium, termed M-MSR (without sucrose, inorganic P, EDTA, calcium, and vitamin sources), was added. The medium was supplemented with 280-μM organic P in the form of Na-phytate (phytic acid sodium salt hydrate, 68388, Sigma-Aldrich, MO, USA). A control treatment was included that consisted only of carrot roots growing in the RC.

### Impact of AM fungal strains and soil types on the bacterial community associated with extraradical hyphae

Bi-compartmented Petri plates were prepared as described above with the AM fungus *R. irregularis* MUCL 43194. Seven weeks after association to the carrot root, the ERH in the RC crossed the partition wall separating the RC from HC and developed profusely in the HC. Two-hundred microliters of bacterial suspension from the five soils described above was inoculated on the surface of the HC. Thus, five treatments and their respective controls (i.e., the HC inoculated with the bacterial suspensions of the five soils without ERH, see Figure S4) were set up with three replicates each. After another 6 days, the M-MSR medium in the HC was dissolved with 35-mL aseptic sodium citrate buffer (10 mM, pH 6) [54], and the hyphae with their surface-associated bacteria were collected. Briefly, the M-MSR medium of the HC was cut into four pieces and distributed equally in two sterile centrifuge tubes of 50 mL, containing 35 mL of sodium citrate buffer, and vortexed for 4 min for dissolution of the phytagel. The hyphae from the two tubes were assembled, forming a pellet, and rinsed gently twice in a Petri plate containing fresh sodium citrate buffer to eliminate the remaining medium. Sterile blotting papers were used to soak up the excess buffer in the hyphal pellet. The controls that contained only the bacterial suspension in the HC were scraped from the surface of the M-MSR medium. The ERH and control samples were collected in 2-mL centrifuge tubes stored at − 80°C for DNA extraction. The total genomic DNA extraction of ERH and M-MSR medium was done using the FastDNA SPIN Kit for Soil (MP Biochemicals, Solon, OH, USA), following the manufacturer’s instructions. PCR amplification of the bacterial 16S rRNA gene V3–V4 region was performed. Pair-end 2 × 250 bp sequencing was performed using the Illumina NovaSeq platform with NovaSeq 6000 SP Reagent Kit (500 cycles) at Shanghai Personal Biotechnology Co., Ltd (see Methods in the Additional file 1).

The same approach as above was conducted with the four AM fungal strains (i.e., *R. irregularis* MUCL 43194, *R*. *irregularis* MUCL 41833, *R*. *clarus* MUCL 46238, and *R*. *intraradices* MUCL 49410) plus one control that consist of carrot roots not associated with an AM fungus, inoculated in the HC with 200 µL bacterial suspension from soil BJ. The BJ soil was selected because it had been collected as part of a long-term experiment with appropriate P application for over 20 years, representing the typical cultivation system of the North China Plain. Three replicates were considered per treatment. After 3 and 6 days, the M-MSR medium in the HC was dissolved with 35-mL sterilized sodium citrate buffer and then processed for DNA extraction as above.

### Isolation and identification of bacteria from extraradical hyphae of *R*. *irregularis* MUCL 43194

Bi-compartmented Petri plates were prepared with carrot roots clone DC2 associated with *R. irregularis* MUCL 43194 as described above. Bacterial suspension of BJ was inoculated in the HC for 6 days. The tiny layers of bacteria that developed around the hyphae were detached carefully with pipette tips under a stereomicroscope at 56 × magnification (Olympus SZX7, Japan). The bacteria were then streaked with the pipette tips on 10-mL solid tryptone soy broth (TSB, CM0129, Thermo Scientific, USA) in 60 × 15 mm plates (for details, see Methods in the Additional file 1). To identify the bacteria, the V3–V4 regions of the 16S rRNA gene were applied on the DNAs extracted using degenerate primers (338F and 806R, Table S8). The PCR products obtained were subsequently sequenced by Sanger sequencing. To recover cultivated bacteria for further studies, the Sanger sequencing results were blasted (BLAST + 2.10.1) to the 16S rRNA gene amplicon sequence variants (ASVs) in the high-throughput sequencing data, allowing to relate the isolated bacteria to relevant ASVs.

### SynCom construction

Bi-compartmented Petri plates were prepared with carrot roots clone DC2 associated with *R. irregularis* MUCL 43194 as described above. Two SynComs were constructed which consist of 20 bacterial isolates (including *Streptomyces* sp. D1) or 19 bacterial isolates (without *Streptomyces* sp. D1). The bacterial isolates were from the ERH of *R. irregularis* MUCL 43194 after inoculation with BJ bacteria suspension (Table S1). Each bacterium was cultured separately in 50-mL tubes, washed, and OD_600_ adjusted to 0.6. The cultures were then mixed in 1:1 ratio and spread onto the HC containing the ERH. Non-mycorrhizal carrot roots plated in the RC and each of the two SynComs inoculated in the HC were considered as controls. After 3 days, the bacterial community on the ERH and on the medium (i.e., the controls) were collected in 2-mL centrifuge tubes stored at − 80°C for DNA extraction. The total genomic DNA was extracted and 16S rRNA gene amplified and analyzed.

In parallel, a similar experiment was conducted in pots under 12-h light–dark cycle at 22°C night and 24°C day (for detailed description of the pot system, see Figure S5). The treatment consisted of the 20 bacterial isolates (thus with *Streptomyces* sp. D1) inoculated in the HC containing the ERH or devoid of ERH (SynCom + AM fungi and SynCom–AM fungi treatments, respectively). Maize (*Zea mays* L., cv. Zhengdan 958) was used as the host plant and *R. irregularis* MUCL 43194 as the AM fungal strain. The soil from Changping, BJ was air-dried, sieved (2 mm), and sterilized by 25 kGy ^60^Co gamma irradiation. The plant compartment was filled with a sterilized mixture of soil and river sand (4:1 v/v). The HC was filled with a sterilized mixture of soil and river sand (1:1, v:v). Ten weeks after planting, the ERH developed into the HC. At that time, the SynCom mixed with Na‐phytate was injected though the tube protruding outside the HC. The solution arrived at the top nylon mesh and then permeated to the soil homogeneously. After another 2 weeks, the soil, river sand, and associated fungal material in the HCs were transferred to a sieve with a 30-μm mesh. The soil was carefully washed through the mesh with filter-sterilized deionized water, leaving the mycelia and river sand on the sieve. To separate the ERH from the river sand and to clean it, the mixture was transferred into a 1-L beaker, and filter-sterilized deionized water was added. The mixture was then stirred gently and poured back into the sieve, leaving the river sand in the beaker. This procedure was repeated five times. The ERH was rinsed with filter-sterilized deionized water before it was collected from the sieve using forceps and placed into 2-mL centrifuge tubes. The control soil samples were also collected in 2-mL centrifuge tubes. The total genomic DNA extraction of ERH and bulk soil was applied for 16S rRNA gene amplification and analysis.

### Quantification of alkaline phosphatase activity of the bacterial isolates

The bacterial isolates were cultured in 1/2 liquid TSB medium at 28°C in the dark. A modified minimal A medium [55–57] was used to measure AP activity (see Methods in the Additional file 1).

### Quantification of alkaline phosphatase activity of *Streptomyces* sp. D1 and *Pseudomonas* sp. H2 in the presence of *R*. *irregularis* MUCL 43194

Zero-point 5-mL cell cultures (*n* = 3) of the two bacteria (for detail, see below — transcriptome landscape of *Streptomyces* sp. D1 and *Pseudomonas* sp. H2 in the presence of *R*. *irregularis* MUCL 43194) were inoculated in the HC in contact with the ERH or in the absence of ERH. After 72 h, the bacteria were collected and resuspended in a Tris–HCl buffer adjusted to pH 9.4 incubated at 30 °C in the dark with 4-mM (final concentration) *para*-nitrophenyl phosphate (*p*NPP). The reaction was stopped using 2-mM (final concentration) NaOH once visible production of the colorimetric product *para*-nitrophenol (*p*NP) was observed. Cell debris and precipitants were removed via centrifugation (3 min, 10,000 g) prior to iMark™ Microplate Reader (optical density 405 nm, Bio-Rad, Hercules, CA, USA). A standard curve for *p*NP was generated using a range of concentrations (0, 2, 4, 12.5, 25, 37.5, 50 µg mL^−1^).

### Transcriptome landscape of *Streptomyces* sp. D1 and *Pseudomonas* sp. H2 in the presence of *R*. *irregularis* MUCL 43194

Transformed carrot roots clone DC2 associated with *R*. *irregularis* MUCL 43194 were grown in bi-compartmented Petri plates as described above with minor modification in the HC. At week 7, the ERH of the AM fungus developed extensively on the slope in the HC. Ten milliliters of liquid M-MSR, containing 280-μM Na‐phytate, was added in the HC allowing the ERH to grow from the slope into the whole HC. After another 4 weeks, the HC was covered by actively growing hyphae. The HC was inoculated with 500-µL *Streptomyces* sp. D1 or *Pseudomonas* sp. H2 at OD_600_ = 0.6. A control that consists of a carrot root without AM fungus grown on the MSR medium was included in the design. Before inoculation, each bacterium was pre-cultured for 16 h in liquid M-MSR medium containing 400-µM inorganic P and then washed three times with M-MSR medium to prevent any excess storage of phosphate that could hinder results. At 3 DAI, bacteria cell cultures in the HC were transferred to a 50-mL tube. After vortexing 4 min, 5 mL of bacteria cell cultures was stored at − 4°C for quantification of AP activity (see above). The rest of bacteria cell cultures was centrifuged (3 min, 10,000 g) to remove medium, and the bacteria cells were stored at − 80°C for RNA extraction (see Methods in the Additional file 1). The transcriptome profile of glucose, fructose, and trehalose metabolism genes in *Streptomyces* sp. D1 was established. The glycolysis, pentose phosphate pathway, citrate cycle, fructose and mannose metabolism, starch and sucrose metabolism, fatty acid biosynthesis, two-component system, pyruvate metabolism, phosphotransferase system, purine metabolism, pyrimidine metabolism terpenoid backbone biosynthesis, sesquiterpenoid triterpenoid biosynthesis, and bacteria secretion system pathways were described in *Streptomyces* sp. D1 or *Pseudomonas* sp. H2, based on KEGG gene annotations.

### Transcriptome landscape of *R*. *irregularis* MUCL 43194 in the presence of *Streptomyces* sp. D1

A similar experimental design as above was followed. The ERH of *R. irregularis* MUCL 43194 was inoculated with *Streptomyces* sp. D1. A control that consists of an AM fungus-colonized carrot root without bacteria was included in the design. At 3 DAI on the ERH, the hyphae were collected, vortexed for 4 min, and stored at − 80°C for RNA extraction (see Methods in the Additional file 1). All the known phosphate-associated genes (including *pho84*, *pho89*, *VTC1/2/4*, *pho80*, *pho85*, *pho81*, *pho4*, *pho91*, *ppn1*; Table S9), some metal ion transporter genes (including *VIT1*, *ZRT1* and *MatA*; Table S9) and energy production genes (including *Idh2*, *NDUFA4*, *ssuE*, and *wrbA*; Table S9) that were significantly differentially expressed based on KEGG gene annotations were described (see Table S9). All the genes to Terms in the Gene Ontology database were mapped and the numbers of differentially enriched genes in each Term calculated. TopGO was used to perform GO enrichment analysis on the genes. *P*-value was calculated by hypergeometric distribution method (the standard of significant enrichment is *P*-value < 0.05). Finally, the GO term with significantly differentially enriched genes was determined to assess the main biological functions performed by the genes.

### Phytate consumption of *Streptomyces* sp. D1 and *Pseudomonas* sp. H2 in the presence of ERH

Transformed carrot roots clone DC2 associated with *R*. *irregularis* MUCL 43194 were grown in bi-compartmented Petri plates (90 × 15 mm) as described above with minor modifications in the HC. At week 7, the ERH of the AM fungus developed extensively on the slope in the HC. Ten milliliters of liquid M-MSR medium, containing 280-μM Na‐phytate, was added in the HC allowing the ERH to grow from the slope into the whole HC. After another 4 weeks, the HC was covered by actively growing hyphae. The HC was inoculated with 500-µL *Streptomyces* sp. D1 or *Pseudomonas* sp. H2 at OD_600_ = 0.6. A control that consists of carrot roots in the RC without AM fungus and thus ERH development in the HC was considered in the design. The two bacterial strains were pre-cultured for 16 h on M-MSR medium containing 400-µM inorganic P and then washed three times with M-MSR medium to prevent any excess storage of phosphate that could hinder results. At 3 DAI, the ERH were collected in sterile 1.5-mL centrifuge tubes and stored at − 80°C for RNA extraction (see Additional file 1). Relative quantitative RT-PCR was performed to quantify the genes expression in hyphae. The ΔCt was calculated by subtracting the Ct value of a reference gene (5.8S rRNA gene) from the Ct value of each target gene. Relative fold-change of each target gene was normalized by the 2^−ΔΔCt^ method, with reference to the ΔCt value in the control. The medium in the HC was passed through an Acrodisc® Syringe Filter (0.2-μm Supor® Membrane, Pall Corporation, New York, USA) to remove the bacterial cells and stored at − 20°C for analysis of inorganic and total P. Inorganic P concentration was determined by the molybdate-blue method [58]. Total P concentration was evaluated by inductively coupled plasma atomic emission spectroscopy (ICP-AES). Phytate-P concentration was calculated by subtracting the inorganic P from the total P concentration. Phytate-P consumption was then calculated with the Phytate-P of each control treatment minus each bacterium inoculation treatment.

### Impact of *Streptomyces* sp. D1 on organic P utilization by *R*. *irregularis* MUCL 43194 and stimulation of gene expression in plants

Three-compartmented pots were constructed, consisting of a central root compartment (RC), separated from an external hyphal compartment (HC) by a buffer compartment (BC) (for detail, see Figure S6). The sides of the two tubes were covered with a nylon mesh of 30-μm pore size (Anping Wire Mesh Industrial Ltd., Hebei, China) preventing the outgrowth of roots from the RC to the BC and HC but allowing the spreading of hyphae in both compartments. The soil from Changping, BJ, was used, air-dried, sieved (2 mm), and sterilized at 25-kGy ^60^Co gamma irradiation. The substrate in the RC and BC was a mixture of river sand and soil, thoroughly rinsed with deionized water, in the ratio of 4:1 (v/v). The HC was filled with a sterilized mixture of river sand and soil (1:1, v:v). A total of 250, 200, and 900 g of substrate was added into the RC, BC, and HC, respectively. Three surface-sterilized seeds of *Medicago truncatula* were placed in the RC. The pots were maintained in a growth chamber under 12-h light–dark cycle at 22°C night and 24°C day. Seven weeks after planting, the hyphae developed into the HC. At that time, the *Streptomyces* sp. D1 or *Pseudomonas* sp. H2 suspension mixed with Na‐phytate was inoculated in the HC. After 7 days, the *M. truncatula* plants were harvested, and roots washed tree times with sterile 1 × PBS buffer and one time with sterile deionized water. The roots were then frozen with liquid nitrogen and preserved at − 80°C for evaluation of the relative expression of genes involved in P transport as well as genes involved in fatty acid biosynthesis, transport, and regulation in roots using relative quantitative RT-PCR.

### Impact of *Streptomyces* sp. D1 exudates on bacteria isolated from ERH of *R*. *irregularis* MUCL 43194

Two-hundred microliters of *Streptomyces* sp. D1 cells suspension (OD_600_ = 0.6) was washed three times in 0.9% NaCl (w:v) solution and incubated on cellophane membranes (35-mm diam., 0.22 µm) in Petri plates (90-mm diam.) containing nutrient broth (0.5% peptone, 0.3% yeast extract, 0.5% NaCl, 0.8% agar). After 5 days, the bacteria covered the cellophane membranes which were gently removed with forceps and placed in the center of larger cellophane membranes (85-mm diam., 0.22 µm) in the middle of Petri plates (90-mm diam.) containing the same nutrient broth medium as above. These membranes were removed after 24 h. The bacterial isolates isolated from the surface of the AM fungal hyphae in contact with the bacterial suspension of soil BJ were incubated at the places where the membranes were removed, using 4 µL of cells suspension (OD_600_ = 0.6). A treatment that consists of cellophane membranes covered with 200 µL of 0.9% NaCl (w:v) solution was included as control. Bacteria were similarly incubated on the medium after the membranes were removed. The diameter of bacterial colonies was measured for both treatments and controls to calculate growth inhibition.

The medium containing nutrient broth was used in pairwise interactions of strains. *Streptomyces* sp. D1 or its zymotic was spotted in the center of Petri plates (90-mm diam.). *Pseudomonas* sp. H2 was inoculated at both sides, 1 cm from the center. Four microliters of cell suspension at an OD_600_ = 0.6 of each stain was used. Four microliters of 0.9% NaCl (w:v) solution or 4-µL TSB liquid medium was inoculated in the center of the Petri plates as the control treatments.

### Identification of antibacterial activity of *Streptomyces* sp. D1

AntiSMASH v.6.0 [59] was used to analyze the genome of *Streptomyces* sp. D1. To detect well-defined clusters containing all secondary metabolism genes, the “strict” detection strictness was chosen in antiSMASH. *Streptomyces* sp. D1 culture broth was extracted with 85% ethanol, evaporated with an N_2_ stream, and dissolved in methanol to analyze metabolites. UPLC-MSMS analysis was performed with an ACQUITY UPLC H-Class system (Waters Alliance, Milford, MA, USA) equipped with a mass spectrometer (API 4000, Applied Biosystems). The UPLC conditions were as follows: flow rate, 0.3 mL min^−1^; solvent A, ammonium acetate water; and solvent B, acetonitrile.

## Statistical analyses

All statistical analyses were conducted in R v.4.0.3 and IBM SPSS Statistics v.21. Co-occurrence networks were constructed based on the relative abundance of ASVs > 0.01%. Robust correlations with Spearman’s correlation coefficients (*ρ*) > 0.65 and false discovery rate-corrected *P*-values < 0.01 were used to construct networks. The networks were visualized with the Fruchterman-Reingold layout with 10^4^ permutations in igraph. Keystone ASVs were identified separately for the − AM fungi and + AM fungi treatments and defined as those nodes within the top 5% of node degree values of each network. The growth curves were fitted by the ggplot2 R package, method = “gam”. For the transcriptome, the filtered reads were mapped to the reference genome using HISAT2 v2.0.5 (Fungi) or Bowtie2 v2.2.6 (bacteria). The HTSeq v0.9.1 statistics were used to compare the read count values on each gene as the original expression of the gene, and FPKM was used to standardize the expression. Then, difference expression of genes was analyzed by DESeq v1.30.0 with screened conditions as follows: expression difference multiple |log2FoldChange|> 1 and significant *P*-value < 0.05. TopGO was used to conduct an analysis of differential genes. The *P*-value was calculated using the hypergeometric distribution method, with a significance threshold set at *P* < 0.05 to determine significant enrichment.

### Supplementary Information

**Additional file 1. **Methods: Biological material - Extraction of bacterial suspensions from soil. Isolation and identification of bacteria from the extraradical hyphae network of *R. irregularis* MUCL 43194. Whole genome sequencing of *Streptomyces* sp. D1 and *Pseudomonas* sp. H2. Competition of *Streptomyces* sp. D1 and *Pseudomonas* sp. H2 in the hyphosphere. Carbon assimilation profiles of bacterial isolates. Pot system set up to study the impact of *Streptomyces* sp. D1 on organic P utilization by *R. irregularis* MUCL 43194 and stimulation of gene expression in plants. Liquid M-MSR medium in the hyphal compartment of the in vitro culture system. Quantification of alkaline phosphatase activity of the bacterial isolates. Competition of *Streptomyces* sp. and *Pseudomonas* sp. on hyphae. Identification of antibacterial activity of *Streptomyces* sp. D1. RNA extraction for RT-PCR and RT-PCR protocol. RNA extraction for transcriptome and transcriptome protocol. DNA extraction. 16S rRNA gene sequence analysis. **Figure S1.** Growth curves of 20 bacterial isolates grown in M9 minimal salts medium with 6 carbon sources (i.e., fructose, glucose, inositol, citric acid, trehalose, or succinic acid) as sole carbohydrate source. Error bars represent the standard deviation of four independent replicates. **Figure S2.** Reconstruction of (A) the *Streptomyces* sp. D1 and (B) *Pseudomonas* sp. H2 major carbohydrate metabolic pathway and phosphate metabolic pathway mapped with transcriptomic data. The reconstructed metabolic pathways of the two bacteria were based on KEGG genes annotations and their relative differential expression profiles of triplicates (each replicate was a mix of 5 culture plates). The genes with a significant (*P* < 0.05) differential expression of >1 |log2FC| were indicated with an arrow (pathway) and a star (heatmap) in green (up-regulated in absence of the extraradical hyphae), red (up-regulated in the presence of the extraradical hyphae). The number in the pathways correspond to the number in the heatmaps. Gray arrow represents the absence of genes identified in the pathway. **Figure S3.** Reconstruction of the *Streptomyces* sp. D1 albaflavenone synthesis pathway mapped with transcriptomic data. The reconstructed metabolic pathway was based on KEGG genes annotations and their relative differential expression profiles of triplicates (each replicate was a mix of 5 culture plates, 15 plates for every treatment). The pathways with genes significant (*P* < 0.05) differential expression of |log2FC| > 1 are indicated with pathway in green (down-regulated in the presence of the extraradical hyphae), red (up-regulated in absence of the extraradical hyphae). **Figure S4.** Bi-compartmented in vitro cultivation system. (A) Ri T-DNA transformed root of carrot developing single in the root compartment (RC) and bacterial community plated in the hyphal compartment (HC), representing the control treatment (RC/HC^–AMF^). (B) Ri T-DNA transformed root of carrot associated with an AM fungus in the RC, with extraradical hyphae (ERH) and spores (in blue) extending in the HC in contact with the bacterial community, representing the ERH treatment (RC/HC^+AMF^). (C) Detailed picture of the bi-compartmented in vitro cultivation system with a carrot root and AM fungus in the RC and ERH crossing the plastic barrier separating the RC from the HC and developing profusely in the HC in contact with a suspension of bacteria. **Figure S5.** Schematic representation of the hyphal compartment (HC). The HC was cuboid (6.8 cm in length, 6.0 cm in width, 5.8 cm in height) and made of PVC plates. In the plate on the top of the HC, a hole (6 mm in diameter) was made and a plastic tube (5 mm in diameter, 50 cm in length) was inserted and fixed with PVC glue. One centimeter below the top plate, another PVC plate with holes (1 mm in diameter) was fixed and covered with a 30 μm nylon mesh. On one side of the HC, a PVC plate with holes (4 mm in diameter) and covered with 30 μm or 0.45 μm nylon mesh was fixed to allow or not the AM fungal hyphae to grow into the HC. Two HCs were buried in the soil at both sides of the pots containing three maize plants associated to an AM fungus. The soil composition in the pot and HCs is detailed (see Methods in Additional file 1). The bacterial suspension was then injected with a syringe through the plastic tube, allowing the bacteria to diffuse homogeneously in the soil of the HC in contact or not with the ERH. **Figure S6.** (A) Schematic representation of the three-compartmented pot experimental system. The inner compartment contains the roots and fungal hyphae (RC), the middle compartment is a buffer compartment (BC) and the outer compartment is the hyphal compartment (HC) allowing only the extraradical hyphae (ERH) to extend. The BC, with a width of 1 cm, was placed between the two compartments to prevent the movement of soluble organic P from HC to RC. A nylon mesh of 30 μm separating the RC from the BC and the BC from the HC was used to avoid roots to extend from RC to HC, while allowing the hyphae to extend from RC to HC. The bacterium was inoculated in the HC in contact with the ERH of *Rhizophagus irregularis *MUCL 43194. Each treatment consisted of four replicates with three *Medicago truncatula* plants per RC. Red lines, extraradical hyphae of *R. irregularis *MUCL 43194; red dots, spores of *R. irregularis *MUCL 43194. (B) Detailed picture of the three-compartmented pot system. **Table S1.** Mean relative abundance of 20 bacterial isolates of all hyphal samples. **Table S2.** Trehalose metabolism and transporter genes in the genome of *Streptomyces* sp. D1 identified with KEGG. **Table S3.** Phosphate metabolism genes in the genome of *Streptomyces* sp. D1 based on KEGG gene annotations. **Table S4.** Phosphate metabolism genes in the genome of *Pseudomonas* sp. H2 based on KEGG gene annotations. **Table S5.** Number of genes significantly differentially expressed for each KEGG metabolic pathways in *Pseudomonas* sp. H2. **Table S6.** Secondary metabolites gene clusters in the genome of *Streptomyces* sp. D1 detected by antiSMASH6 pipeline. **Table S7.** Physico-chemical properties of the soils used to extract bacterial suspensions. **Table S8.** Summary of primers used in the study. **Table S9.** Phosphate, polyphosphate and associated genes expression significantly increased in *Rhizophagus* *irregularis* MUCL 43194 inoculated with *Streptomyces* sp. D1 as compared to the fungus grown in absence of the bacteria.

**Additional file 2. **CAZy genes in D1 and CAZy genes in H2.

**Additional file 3. **Flagellar assembly genes in D1 and Flagellar assembly genes in H2.

## Data Availability

Sequences data that support the findings of this study (amplicon sequencing, RNA-seq, and genome sequencing) were deposited in the NCBI SRA database with SRA accession codes SRR23819454 and SRR23819565 (BioSample: SAMN33726247) and BioProject code PRJNA943439.
