# Supplementary material for: Arbuscular mycorrhizal fungi and Streptomyces: brothers in arms to shape the structure and function of the hyphosphere microbiome in the early stage of interaction
Source: Microbiome. 2024 May 9;12:83. doi: 10.1186/s40168-024-01811-2 (PMC11080229; doi:10.1186/s40168-024-01811-2)
Supplement: Supplementary file 2 — Additional file 1. Methods: Biological material - Extraction of bacterial suspensions from soil. Isolation and identification of bacteria from the extraradical hyphae network of R. irregularis MUCL 43194. Whole genome sequencing of Streptomyces sp. D1 and Pseudomonas sp. H2. Competition of Streptomyces sp. D1 and Pseudomonas sp. H2 in the hyphosphere. Carbon assimilation profiles of bacterial isolates. Pot system set up to study the impact of Streptomyces sp. D1 on organic P utilization by R. irregularis MUCL 43194 and stimulation of gene expression in plants. Liquid M-MSR medium in the hyphal compartment of the in vitro culture system. Quantification of alkaline phosphatase activity of the bacterial isolates. Competition of Streptomyces sp. and Pseudomonas sp. on hyphae. Identification of antibacterial activity of Streptomyces sp. D1. RNA extraction for RT-PCR and RT-PCR protocol. RNA extraction for transcriptome and transcriptome protocol. DNA extraction. 16S rRNA gene sequence analysis. Figure S1. Growth curves of 20 bacterial isolates grown in M9 minimal salts medium with 6 carbon sources (i.e., fructose, glucose, inositol, citric acid, trehalose, or succinic acid) as sole carbohydrate source. Error bars represent the standard deviation of four independent replicates. Figure S2. Reconstruction of (A) the Streptomyces sp. D1 and (B) Pseudomonas sp. H2 major carbohydrate metabolic pathway and phosphate metabolic pathway mapped with transcriptomic data. The reconstructed metabolic pathways of the two bacteria were based on KEGG genes annotations and their relative differential expression profiles of triplicates (each replicate was a mix of 5 culture plates). The genes with a significant (P < 0.05) differential expression of >1 |log2FC| were indicated with an arrow (pathway) and a star (heatmap) in green (up-regulated in absence of the extraradical hyphae), red (up-regulated in the presence of the extraradical hyphae). The number in the pathways correspond to the nu [file 40168_2024_1811_MOESM1_ESM.docx]

**Additional file 1**

**Methods**

**Biological material - Extraction of bacterial suspensions from soil.** A stock solution of Nycodenz (Nycodenz AG, 1002424, Alere Technologies AS, Oslo, Norway) was prepared by dissolving 8 g of Nycodenz in 10 mL water and sterilizing it by filtration. A solution of Tween80/Tetra Sodium Pyrophosphate (TTSP) was also prepared to a final concentration of 50 mM tetrasodium pyrophosphate and 0.05% Tween 80 in 500 mL water and sterilizing it by filtration. Four mL of TTSP was added to 2 g of each soil in 10 mL centrifuge tubes and vortexed for 1 min. One mL of the liquid phase was transferred to 2 mL centrifuge tubes containing 500 µL of Nycodenz. The tubes were centrifuged at 10,000 g for 10 min. After centrifugation, 1 mL of the upper and middle phase of the solution was transferred to 10 mL centrifuge tubes containing 6 mL of liquid sterilized (121°C for 15 min) M-MSR medium. After shaking, the tubes were centrifuged at 5000 g for 10 min at 4°C. The supernatants were discarded and the bacterial cells re-suspended in 1 mL of sterilized M-MSR medium in 10 mL centrifuge tubes.

**Isolation and identification of bacteria from the extraradical hyphae network of *R. irregularis*** **MUCL 43194.** The tiny layers of bacteria that wraps around the hyphae were detached carefully with pipette tips under a stereomicroscope at 56 × magnification. The bacteria were then streaked with the pipette tips on Petri plates (60 × 15 mm) containing 10 mL solid Tryptone Soy Broth (TSB, CM0129, Thermo Scientific, US) medium. Briefly, the bacteria on and in the sterile pipette tip were streaked in three parallel lines. The operation was repeated with a second and third pipette tip containing bacteria spread out in two other sections of the Petri plate [1]. After 3 to 5 d of incubation in a growth chamber set at 28°C in the dark, single colonies were visible and again picked with a pipette and streaked in Petri plates on fresh TSB medium. This process was repeated three times in total. Single colonies of bacteria were then incubated in 50 mL flasks containing 20 mL liquid 1/2 TSB and incubated at 28°C and 180 rpm in the dark for 5 to 7 d. When the bacterial solution was turbid, 6 µL of the bacterial cultures were transferred into a PCR tube for DNA extraction (see below). To identify the bacteria, the V3–V4 regions of the 16S rRNA gene was applied on the DNAs extracted using degenerate primers (338F and 806R, Table S8). The PCR products obtained were subsequently sequenced by Sanger. To recover the cultivated bacteria for other experiments, the Sanger sequencing results were blasted (BLAST+ 2.10.1) to the 16S rRNA gene Amplicon Sequence Variants (ASVs) in the high-throughput sequencing results of AM fungal strains and soil types. These ASVs were associated with the extraradical hyphae (ERH) of four different arbuscular mycorrhizal (AM) fungi, namely *R*. *irregularis* MUCL 43194, *R*. *irregularis* MUCL 41833, *R*. *clarus* MUCL 46238, and *R*. *intraradices* MUCL 49410. The purpose was to establish a connection between the isolated bacteria and relevant taxa, even when the fungi were developing in the absence of ERH. Sixty-two bacterial isolates were isolated from the surface of ERH of *R*. *irregularis* MUCL 43194. The isolated bacteria were further preserved at –80°C in cryopreservation tubes containing 800 μL of bacterial solution and an equal volume of 80% sterilized (121°C for 15 min) glycerol. The 16S rRNA gene V3-V4 region sequences of these bacteria were used to reconstruct phylogenetic trees by using the maximum-likelihood (ML) method with 1000 bootstrap replications in MEGA version 7.0. Twenty isolates (Table S1) were selected for further study by clustering with 97% similarity to eliminate redundant sequences.

**Whole genome sequencing of *Streptomyces* sp. D1 and *Pseudomonas* sp. H2.** Total DNA extraction of *Streptomyces* sp. D1 and *Pseudomonas* sp. H2 was done via CTAB methods. The genomes were sequenced using the PacBio Sequel and illumina Novaseq platforms (Shanghai Personal Biotechnology Co., Ltd., China). The PacBio sequence reads were assembled with HGAP4 and CANU (Version 1.6), corrected by Illumina Novaseq with pilon (Version 1.22). The KO and pathway annotations of protein coding genes were mainly based on the KAAS (KEGG Automatic Annotation Server, Moriya Y, 2007), in which the gene set is selected as "For Eukaryotes" and the discriminant rule of gene KO was selected as bi-directional best hit (BBH).

**Competition of *Streptomyces* sp. D1 and *Pseudomonas* sp. H2 in the hyphosphere.** Bi-compartmented Petri plates were prepared with carrot roots clone DC2 associated to *R*. *irregularis* MUCL 43194 as described above. *Streptomyces* sp. D1 and *Pseudomonas* sp. H2 were first grown during 5 days at 28°C in 50 mL flasks containing 20 mL 1/2 liquid TSB. Five mL culture of each isolate was then centrifuged in 10 mL centrifuge tubes at 10,000 g for 3 min to remove the growth medium. Five mL fresh M-MSR medium was added to resuspend tubes by shaking. The tubes were centrifuged for another 3 min at 10,000 g. This process was repeated three times and bacteria were resuspended in M-MSR medium to a starting OD_600_=0.6. The HC was inoculated with either 500 µL *Streptomyces* sp. D1 (OD_600_=0.6), 500 µL *Pseudomonas* sp. H2 (OD_600_=0.6), or with both bacteria at 250 µL each. A treatment that consist of carrot roots in the RC without AM fungus and thus ERH development in the HC was considered as control. For the single isolate treatments, the bacteria were gently spread over the surface of the HC with sterile spreaders. For the combined treatment, each isolate with OD_600_=0.6 was combined and spread in the HC. Samples were collected after 3 days. Total genomic DNA extraction was conducted (see Methods in the Additional file 1). Four replicates were considered per treatment (for analysis details, see Methods in the Additional file 1).

**Carbon assimilation profiles of bacterial isolates.** The bacterial isolates stored at –80°C were reactivated in 1/2 liquid TSB at 28°C in the dark and tested for growth performance on 6 Carbon (C) sources reported in the hyphal exudates of AM fungi [2-4]. Bacteria were grown in 100-microwell plates containing 200 μL 1×M9 minimal salts medium [5] (KH_2_PO_4_ 3 g L^−1^; NaCl 0.5 g L^−1^; Na_2_HPO_4_ 6.78 g L^−1^; NH_4_Cl 1 g L^−1^), supplemented with 2 mM MgSO_4_, 0.1 mM CaCl_2_, 10 μM FeSO_4_ [6], and one of the following C sources: 10 mM of fructose, glucose, inositol, citric acid, 5 mM trehalose, or 15 mM succinic acid. Growth was monitored during 48 h by measuring optical density (OD at 600 nm) using a Bioscreen C MBR (Oy Growth Curves Ab Ltd, Helsinki, Finland). Each treatment was replicated four times, and the OD_600_ values determined every 30 min and reported as the mean of five measurements.

**Microcosm experiment**. Spores of *R*. *irregularis* MUCL 43194 were produced *in vitro* on Ri- T-DNA transformed root organs of carrot [7]. After 6 months, numerous spores were obtained and harvested by blending the M-MSR medium containing the spores two times for 4 min in 35 ml of 10 mM sodium citrate, pH 6.0. The supernatant was filtered through a 50 µm mesh to collect the spores before resuspending in sterile deionized H_2_O. In parallel, maize seeds were sterilized with 10% (v/v) H_2_O_2_ for 10 min and 70% (v/v) ethanol for 3 min and then rinsed eight times with sterile deionized water. After soaking the seeds in water at 27°C in the dark for 2 d, three maize seeds were placed in pots (Figure S5).

The system consisted of a pot containing three maize plants associated to an AM fungus and two hyphal compartments (HC) in which bacterial suspensions were injected via a tube (see detailed schema of the HC in Figure S5). The pot had a diameter of 31 cm (top) and 21 cm (bottom) and a height of 26 cm, with a total capacity of 10 kg of soil (RC). The HC was cuboid (6.8 cm in length, 6.0 cm in width, 5.8 cm in height). The following nutrients were added to one kg of soil of Beijing and used in the RC (soil Changping, BJ, see Table S7): 25 mg P (KH_2_PO_4_), 300 mg N (NH_4_SO_4_), 300 mg K (K_2_SO_4_), 75 mg Mg (MgSO_4_·7H_2_O), 7.5 mg Zn (ZnSO_4_·7H_2_O), 7.5 mg Mn (MnSO_4_·H_2_O), and 7.5 mg Cu (CuSO_4_·5H_2_O), and 1% nitrification inhibitor (3,4-dimethylpyrazole phosphate, Sinopharm Chemical Reagent Co. Ltd., Shanghai, China). Each HC received 160 g soil containing 100 mg kg^−1^ organic P in form of Na‐phytate (Phytic acid sodium salt hydrate, 68388, Sigma-Aldrich, MO, US). Two HCs were placed in each pot as follows: first, 2 kg of soil was added on the bottom of the pot. Second, the two HCs were placed horizontally on the surface of the soil, with the nylon mesh oriented toward the center of the pot. Then 7 kg of soil was added to the pot to cover the HCs. Above the buried HCs, 1500 spores of *R.* *irregularis* MUCL 43194 were pipetted, and a thin layer of soil was added to cover the inoculant. Three pre-germinated seeds were placed, and the rest of the soil was added.

**Pot system set up to study the impact of *Streptomyces* sp. D1 on organic P utilization by *R. irregularis* MUCL 43194 and stimulation of gene expression in plants.** The pots had a diameter of 13 cm (top) and 9 cm (bottom) and a height of 10 cm. The root compartment (RC) was delimited by a PVC tube of 10 cm in height and 5 cm in diameter. This PVC tube was placed in a second PVC tube, slightly larger (7.5 cm diameter), forming the buffer compartment (BC) separating the root compartment (RC) from the hyphal compartment (HC) (See Figure S6). The following nutrients were added per kg of substrate in the RC and HC: 20 mg P (KH_2_PO_4_), 300 mg N (NH_4_SO_4_), 300 mg K (K_2_SO_4_), 75 mg Mg (MgSO_4_·7H_2_O), 7.5 mg Zn (ZnSO_4_·7H_2_O), 7.5 mg Mn (MnSO_4_·H_2_O), and 7.5 mg Cu (CuSO_4_·5H_2_O), to facilitate normal growth of *M. truncatula.* One % nitrification inhibitor (3,4-dimethylpyrazole phosphate, Sinopharm Chemical Reagent Co. Ltd., Shanghai, China) was added to the RC. Two hundred gram of substrate was added into the RC and topped with 5 ml suspension containing approximately 600 spores and carrot root fragments colonized by *R. irregularis* MUCL 43194 isolated from *in vitro* bi-compartmented Petri plates. Then, 50 g of substrate was added on top of the layer of spores. Each HC received 900 g soil containing 50 mg kg^−1^ organic P in form of Na‐phytate (Phytic acid sodium salt hydrate, 68388, Sigma-Aldrich, MO, US).

**Liquid M-MSR medium in the hyphal compartment of the *in vitro* culture system.** Transformed carrot roots clone DC2 and *R*. *irregularis* MUCL 43194 were grown in bi-compartmented Petri plates (90 × 15 mm) (see Figure S4) with minor modification in the HC. At week 7, the ERH of AM fungus developed extensively on the slope in the HC (Figure S4). Ten mL of liquid M-MSR, containing 280 μM Na‐phytate, was added in the HC allowing the ERH to grow from the slope into the whole HC. After another 4 weeks, the HC was covered by actively growing hyphae.

**Quantification of alkaline phosphatase activity of the bacterial isolates.** The medium comprised glucose 10 mM, NaCl 200 mg L^−1^, NH_4_Cl 450 mg L^−1^, CaCl_2_ 200 mg L^−1^, KCl 200 mg L^−1^, MgCl_2_ 450 mg L^−1^, with trace metals FeCl_2_ 10 mg L^−1^ and MnCl_2_ 10 mg L^−1^, with 10 mM 4-(2-hydroxyethyl)-1-piperazineethanesulfonic acid (HEPES) buffer at pH 7. KH_2_PO_4_ was then added to a final concentration of 50 µM or 400 µM. Each bacterial isolate was pre-cultured 16 h on minimal A medium containing 400 µM Pi to prevent any excess storage of phosphate that could hamper the results. Five hundred µL pre-cultured inoculant (OD_600_=0.6) of each bacterial isolate was added to 5 mL minimal A medium containing 50 µM Pi in 50 mL centrifuge tubes. After cultivation in Pi-depleted (50 µM) conditions at 28°C in the dark for 48 h, a 0.5 mL culture (n = 5) was incubated with 20 µL para-nitrophenyl phosphate (pNPP) (final conc. 4 mM) and incubated at 30℃ for 1 h. Cell debris and precipitants were removed via centrifugation (3 min, 10,000 g) prior to iMarkTM Microplate Reader (optical density 405 nm, Bio-Rad, Hercules, CA, USA). A standard curve for para-nitrophenol was generated using a range of concentrations (0, 10, 20, 30, 40, 60, 80 µg mL^−1^). Alkaline phosphatase production efficiency was calculated based on per unit carbon input and per unit OD_600_ values of each bacterial isolate.

**Competition of *Streptomyces* sp. and *Pseudomonas* sp. on hyphae.** One µL of DNA from hyphae or M-MSR medium samples was used as template for real-time PCR and two technical replicates were utilized for each sample. The qPCR was performed using a Bio-Rad CFX96 real-time detection system (CA, USA) and the SYBR Green Premix Ex Taq (Takara Bio, RR820A, Japan). Two pairs of lineage-specific primers were used (StF and StR, PsF and PsR; see Table S8). Standard curves were generated by amplification of serial 4 dilutions of the standard plasmid DNA template (in duplicate), with a maximum concentration of 0.005 ng μL^−1^. The absolute abundance of *Streptomyces* and *Pseudomonas* in ERH samples and M-MSR medium samples were thus quantified by using the standard curves (*R*^2^ = 0.99).

**Identification of antibacterial activity of *Streptomyces* sp. D1.** Two hundred µL of *Streptomyces* sp. D1 cells suspension (OD_600_=0.6) was washed three times in 0.9% normal saline solution and incubated on cellophane membranes (35 mm diam., 0.22 µm) in Petri plates (90 mm diam.) containing nutrient broth (0.5% peptone, 0.3% yeast extract, 0.5% NaCl, 0.8% agar). After 5 days, the bacteria covered the cellophane membranes. These were gently removed with forceps and placed in the center of larger cellophane membranes (85 mm diam., 0.22 µm) in the middle of Petri plates (90 mm diam.) containing the same nutrient broth medium as above. These membranes were removed after 24 h. The bacterial isolates isolated from the surface of the AM fungus hyphae in contact with the bacterial suspension of soil BJ were incubated at the places where the membranes were removed, using 4 µL of cells suspension (OD_600_ = 0.6). A control treatment that consists of cellophane membranes covered with 200 µL of 0.9% NaCl (w:v) solution was included. Bacteria were similarly incubated on the medium for 6 days. The effect of the exudates of *Streptomyces* sp. D1 on bacteria growth inhibition was assessed as follows:

$$Growth inhibition \left( \% \right)=\frac{Dc-Da}{\mathrm{Dc}}\times100$$

Dc = diam of the control = growth of the bacteria in absence of exudates of *Streptomyces* sp. D1.

Da = diam of the bacteria in presence of exudates of *Streptomyces* sp. D1.

**RNA extraction for RT-PCR and RT-PCR protocol.** The total RNA of AM fungi hyphal pellets and *Medicago* *truncatula* roots were extracted using RNeasy Plant Mini Kit (Qiagen), according to the manufacturer’s instructions. For single-strand cDNA synthesis, 110 ng of total RNA in roots or 15 ng of total RNA in hyphae was reverse-transcribed at 42°C for 2 min, 37°C for 15 min and 85°C for 5 s in a final volume of 20 μl using PrimeScript™ RT Reagent Kit with gDNA Eraser (Catalogue number: RR047A; TaKaRa Bio Inc, Japan). The products were then diluted to 100 μl. Relative quantitative real-time PCR was performed using a CFX96™ Real-Time System (BIO-RAD, America). Each PCR reaction was conducted in a total volume of 25 μl containing 2 μl cDNA, 13 μl TB Green® Premix Ex Taq™ II (Catalogue number: RR820A, TaKaRa Bio Inc, Japan), 1 μl of each primer (10 μM) and 9 μl ddH2O. The following PCR program was run: 95°C for 300 s, 40 cycles of 95°C for 15 s, 52°C for 15 s and 72°C for 45 s with roots and 95°C for 300 s, 40 cycles of 95°C for 10 s, 60°C for 30 s and 72°C for 45 s with hyphae.

**RNA extraction for transcriptome and transcriptome protocol.** Total RNA was extracted using Trizol Reagent (Invitrogen Life Technologies) and assessed for concentration, quality, and integrity with a NanoDrop spectrophotometer (Thermo Scientific). Three micrograms of RNA were used for library preparation. Initially, mRNA was isolated from total RNA using poly-T oligo-attached magnetic beads. RNA was then fragmented in Illumina proprietary buffer at high temperatures with divalent cations. First-strand cDNA synthesis was carried out using random oligonucleotides and Super Script III, followed by second-strand cDNA synthesis using DNA Polymerase I and RNase H. Blunt ends were generated by exonuclease activities, and the enzymes were subsequently removed. Adenylation of the DNA fragments 3’ ends were performed before ligation of Illumina PE adapter oligonucleotides. The library fragments were purified to select sizes of 400-500 bp using the AMPure XP system (Beckman Coulter, Beverly, CA, USA). DNA fragments with adaptors on both ends were enriched through a 15-cycle PCR reaction with the Illumina PCR Primer Cocktail, followed by purification (AMPure XP system) and quantified using Agilent high sensitivity DNA assay on a Bioanalyzer 2100 system (Agilent). The completed sequencing library was then sequenced on a NovaSeq 6000 platform (Illumina) by Shanghai Personal Biotechnology Co., Ltd.

Transcriptome analysis flow: Quality control begins with sequencing the samples to obtain image files. These are transformed by the sequencing platform’s software to produce FASTQ format raw data. Due to the presence of connectors and low-quality reads in the data, we employ Cutadapt (v1.15) to filter and obtain high-quality sequences for further analysis. The reads mapping is executed using HISAT2 (v2.0.5) for fungi or Bowtie2 (v2.2.6) for bacteria on the reference genome. For differential expression analysis, HTSeq (v0.9.1) is used to calculate read count values followed by FPKM normalization. DESeq (v1.30.0) analyzes gene expression differences with criteria set at |log2FoldChange| > 1 and P-value < 0.05.

**DNA extraction.** The total genomic DNA extraction of ERH and M-MSR medium was achieved using the FastDNA SPIN Kit for Soil (MP Biochemicals, Solon, OH, USA), following the manufacturer’s instructions, and stored at -80 °C prior to further analysis. The quantity and quality of extracted DNAs were measured using a NanoDrop NC2000 spectrophotometer (Thermo Fisher Scientific, Waltham, MA, USA) and agarose gel electrophoresis.

For bacteria identification, the DNA extraction of the bacterial cultures was as follows: 10 µL lysis buffer was added to the 6 µL bacterial culture, mixed with a pipette and incubated in a PCR machine at 95°C for 30 min. The tubes were then cooled to room temperature and 10 µL of neutralization buffer was added to each PCR tube. The final products were used as PCR template. The alkaline lysis buffer (pH=12) and the neutralization buffer (pH=7.5) were prepared as previously described [1].

**16S rRNA gene sequence analysis.** PCR amplification of the bacterial 16S rRNA gene V3–V4 region was performed using the forward primer 338F (5'-ACTCCTACGGGAGGCAGCA-3') and the reverse primer 806R (5'GGACTACHVGGGTWTCTAAT-3'). Sample-specific 7-bp barcodes were incorporated into the primers for multiplex sequencing. The PCR amplicons were purified with Vazyme VAHTSTM DNA Clean Beads (Vazyme, Nanjing, China) and quantified using the Quant-iT PicoGreen dsDNA Assay Kit (Invitrogen, Carlsbad, CA, USA). After the individual quantification step, amplicons were pooled in equal amounts, and pair-end 2 × 250 bp sequencing was performed using the Illlumina NovaSeq platform with NovaSeq 6000 SP Reagent Kit (500 cycles) at Shanghai Personal Biotechnology Co., Ltd (Shanghai, China). Microbiome bioinformatics were performed with QIIME2 2019.4 [8] with slight modifications according to the official tutorials (https://docs.qiime2.org/2019.4/tutorials/). Briefly, raw sequence data were demultiplexed using the demux plugin followed by primers cutting with cutadapt plugin [9]. Sequences were then quality-filtered, denoised, merged and chimera removed using the DADA2 plugin [10]. Non-singleton amplicon sequence variants (ASVs) were aligned with mafft [11]. Taxonomy was assigned to ASVs using the classify-sklearn Naive Bayes taxonomy classifier in feature-classifier plugin [12] against the SILVA Release 132 Database [13].

**Figures**

**
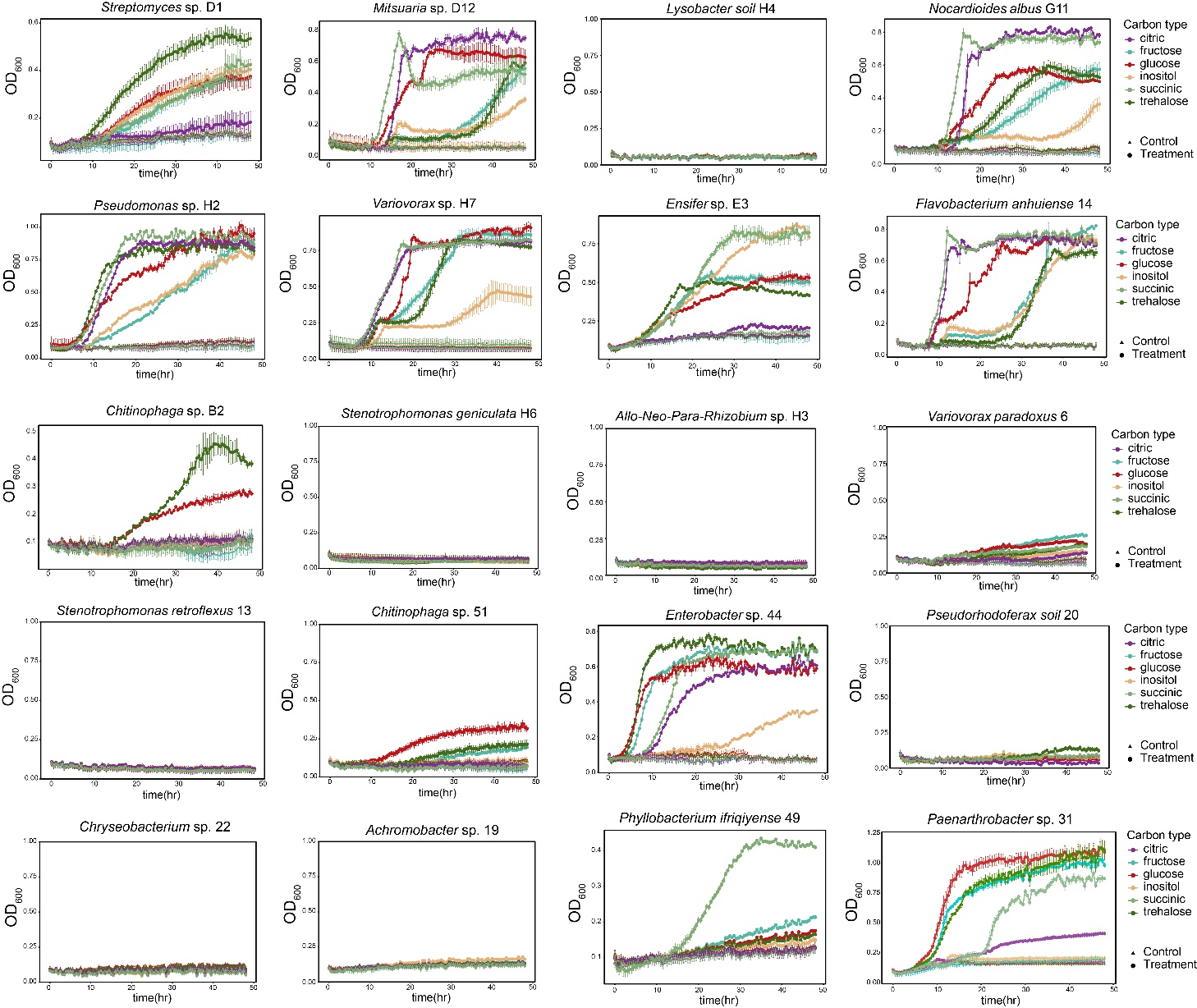
**

**Figure S1** Growth curves of 20 bacterial isolates grown in M9 minimal salts medium with 6 carbon sources (i.e., fructose, glucose, inositol, citric acid, trehalose, or succinic acid) as sole carbohydrate source. Error bars represent the standard deviation of four independent replicates.

**
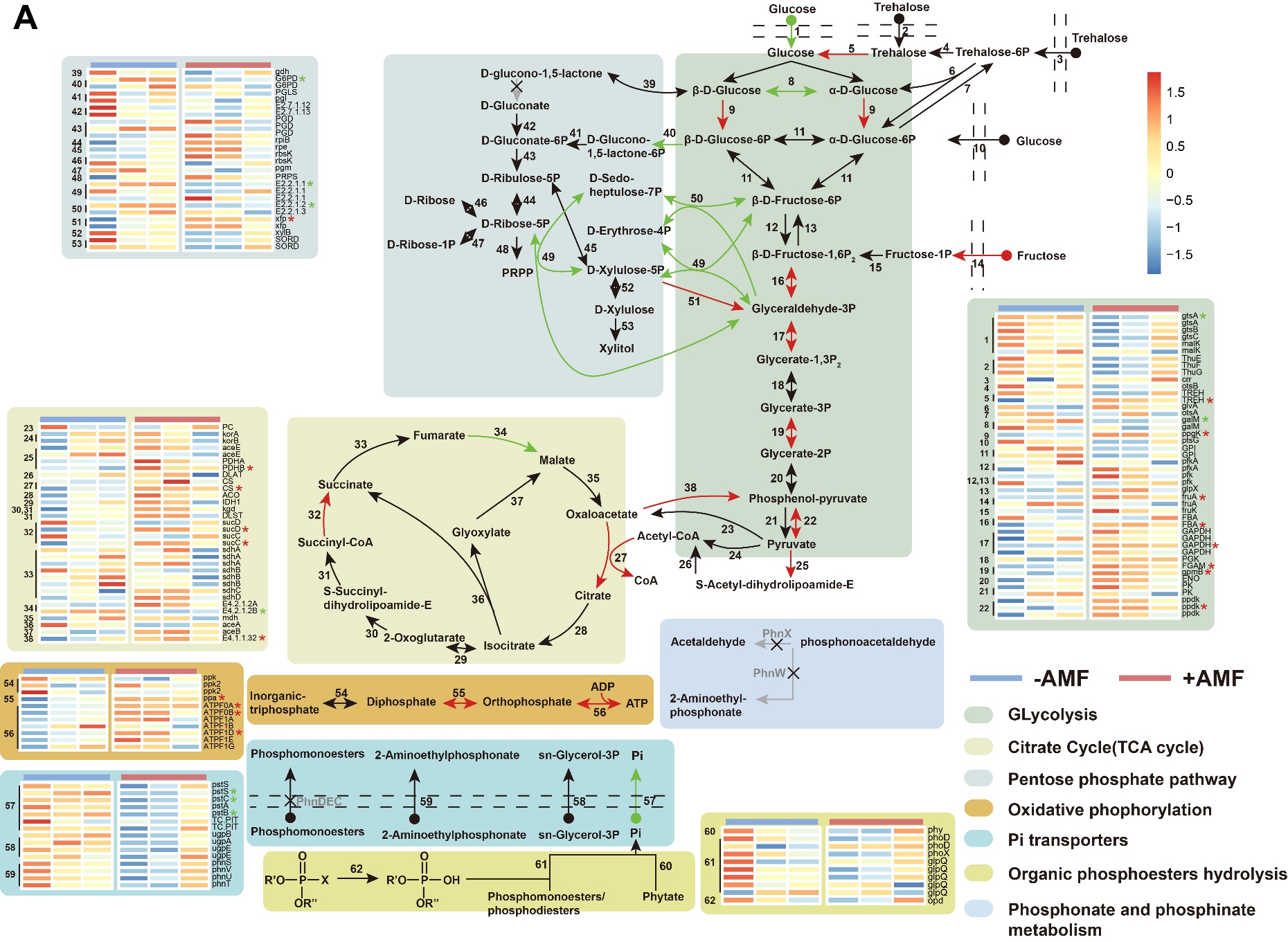
**

**
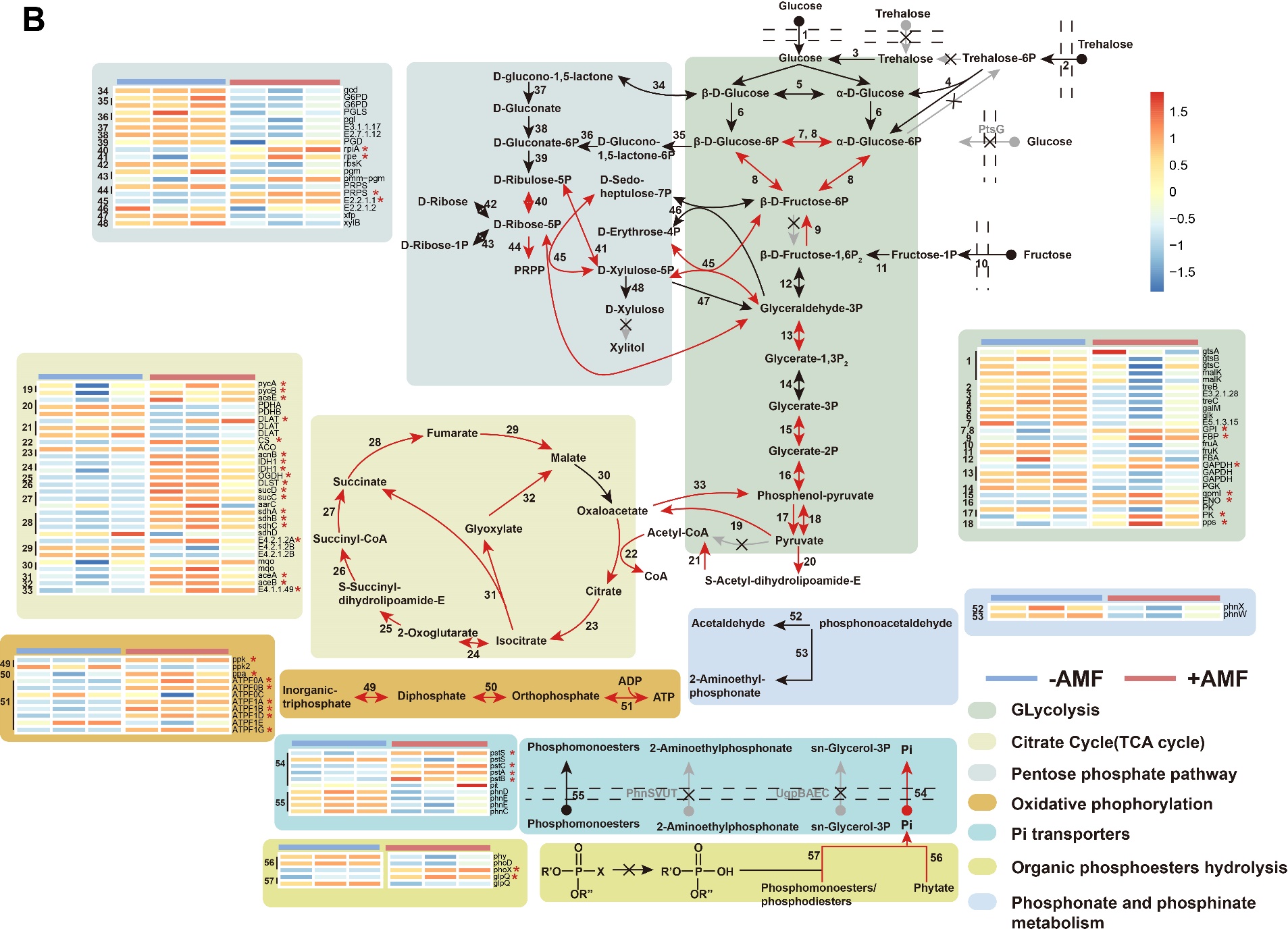
**

**Figure S2** Reconstruction of (**A**) the *Streptomyces* sp. D1 and (**B**) *Pseudomonas* sp. H2 major carbohydrate metabolic pathway and phosphate metabolic pathway mapped with transcriptomic data. The reconstructed metabolic pathways of the two bacteria were based on KEGG genes annotations and their relative differential expression profiles of triplicates (each replicate was a mix of 5 culture plates). The genes with a significant (*P* < 0.05) differential expression of >1 |log2FC| were indicated with an arrow (pathway) and a star (heatmap) in green (up-regulated in absence of the extraradical hyphae), red (up-regulated in presence of the extraradical hyphae). The number in the pathways correspond to the number in the heatmaps. Gray arrow represents the absence of genes identified in the pathway.


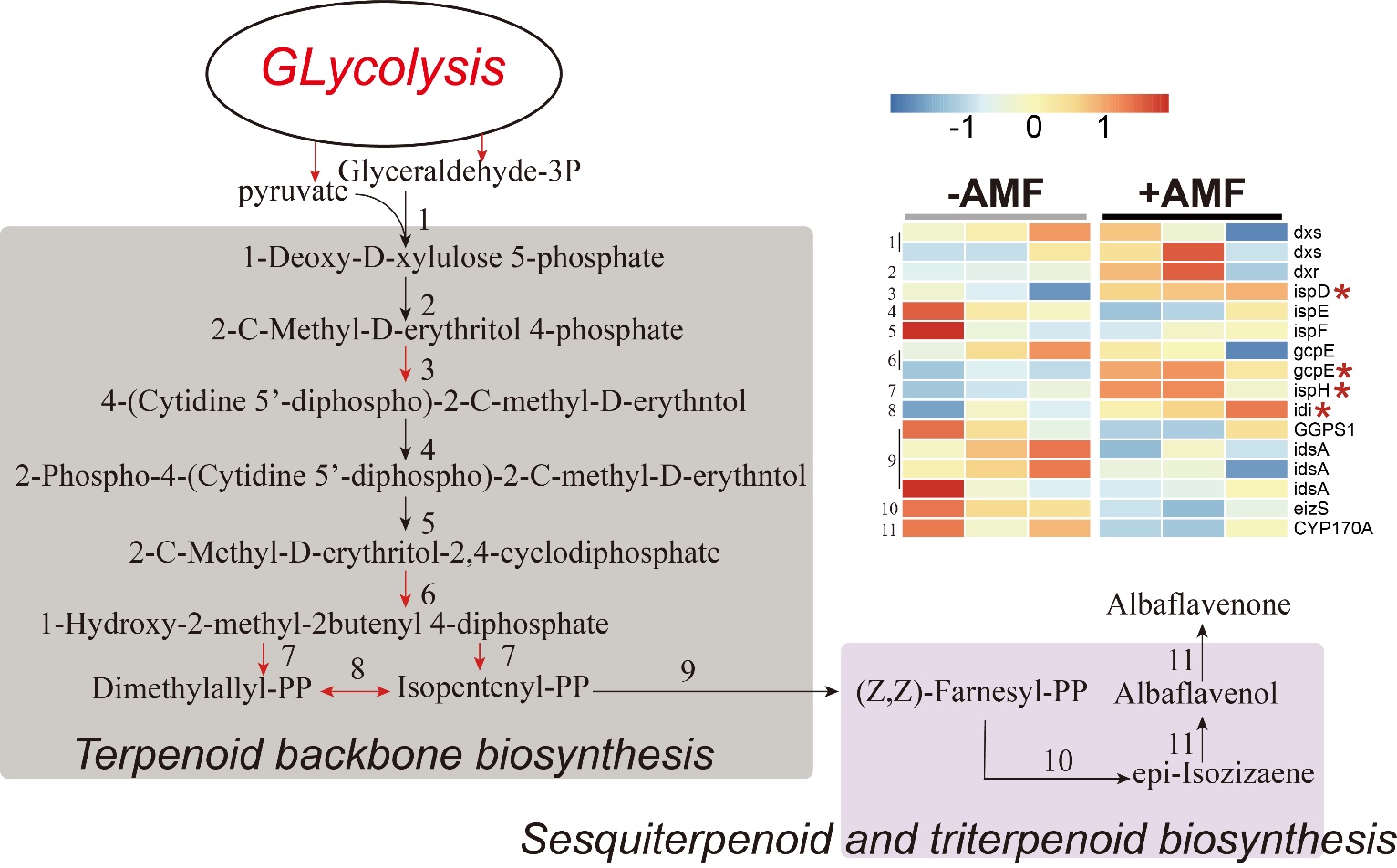


**Figure S3** Reconstruction of the *Streptomyces* sp. D1 albaflavenone synthesis pathway mapped with transcriptomic data. The reconstructed metabolic pathway was based on KEGG genes annotations and their relative differential expression profiles of triplicates (each replicate was a mix of 5 culture plates, 15 plates for every treatment). The pathways with genes significant (*P* < 0.05) differential expression of |log2FC| > 1 are indicated with pathway in green (down-regulated in presence of the extraradical hyphae), red (up-regulated in absence of the extraradical hyphae).

**
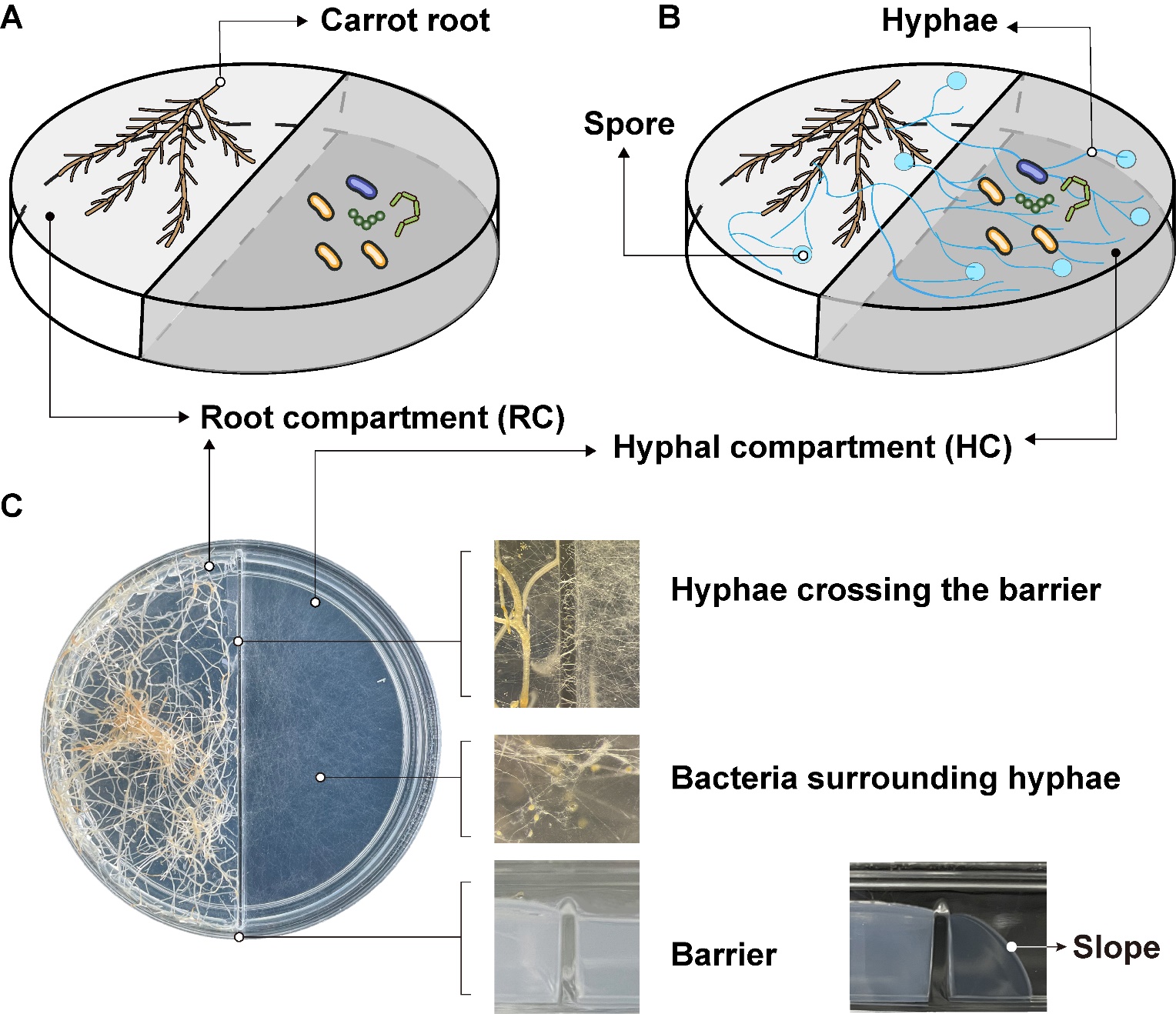
**

**Figure S4** Bi-compartmented *in vitro* cultivation system. (**A**) Ri T-DNA transformed root of carrot developing single in the root compartment (RC) and bacterial community plated in the hyphal compartment (HC), representing the control treatment (RC/HC^–AMF^). (**B**) Ri T-DNA transformed root of carrot associated with an AM fungus in the RC, with extraradical hyphae (ERH) and spores (in blue) extending in the HC in contact with the bacterial community, representing the ERH treatment (RC/HC^+AMF^). (**C**) Detailed picture of the bi-compartmented *in vitro* cultivation system with a carrot root and AM fungus in the RC and ERH crossing the plastic barrier separating the RC from the HC and developing profusely in the HC in contact with a suspension of bacteria. AMF, arbuscular mycorrhizal fungus.

**
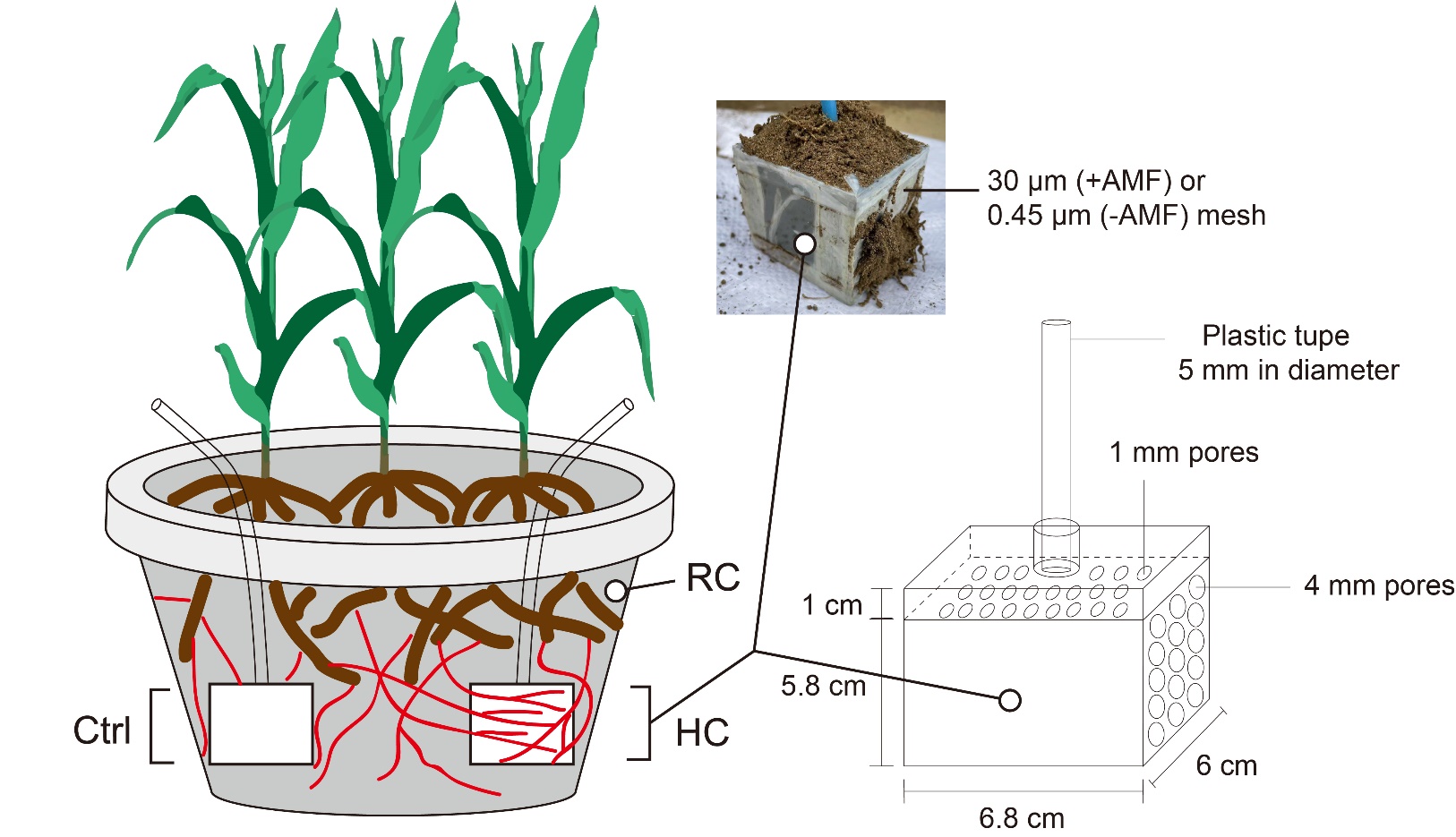
**

**Figure S5** Schematic representation of the hyphal compartment (HC). The HC was cuboid (6.8 cm in length, 6.0 cm in width, 5.8 cm in height) and made of PVC plates. In the plate on the top of the HC, a hole (6 mm in diameter) was made and a plastic tube (5 mm in diameter, 50 cm in length) was inserted and fixed with PVC glue. One centimeter below the top plate, another PVC plate with holes (1 mm in diameter) was fixed and covered with a 30 μm nylon mesh. On one side of the HC, a PVC plate with holes (4 mm in diameter) and covered with 30 μm or 0.45 μm nylon mesh was fixed to allow or not the AM fungal hyphae to grow into the HC. Two HCs were buried in the soil at both sides of the pots containing three maize plants associated to an AM fungus. The soil composition in the pot and HCs is detailed (see Methods in Additional file 1). The bacterial suspension was then injected with a syringe through the plastic tube, allowing the bacteria to diffuse homogeneously in the soil of the HC in contact or not with the ERH.

**
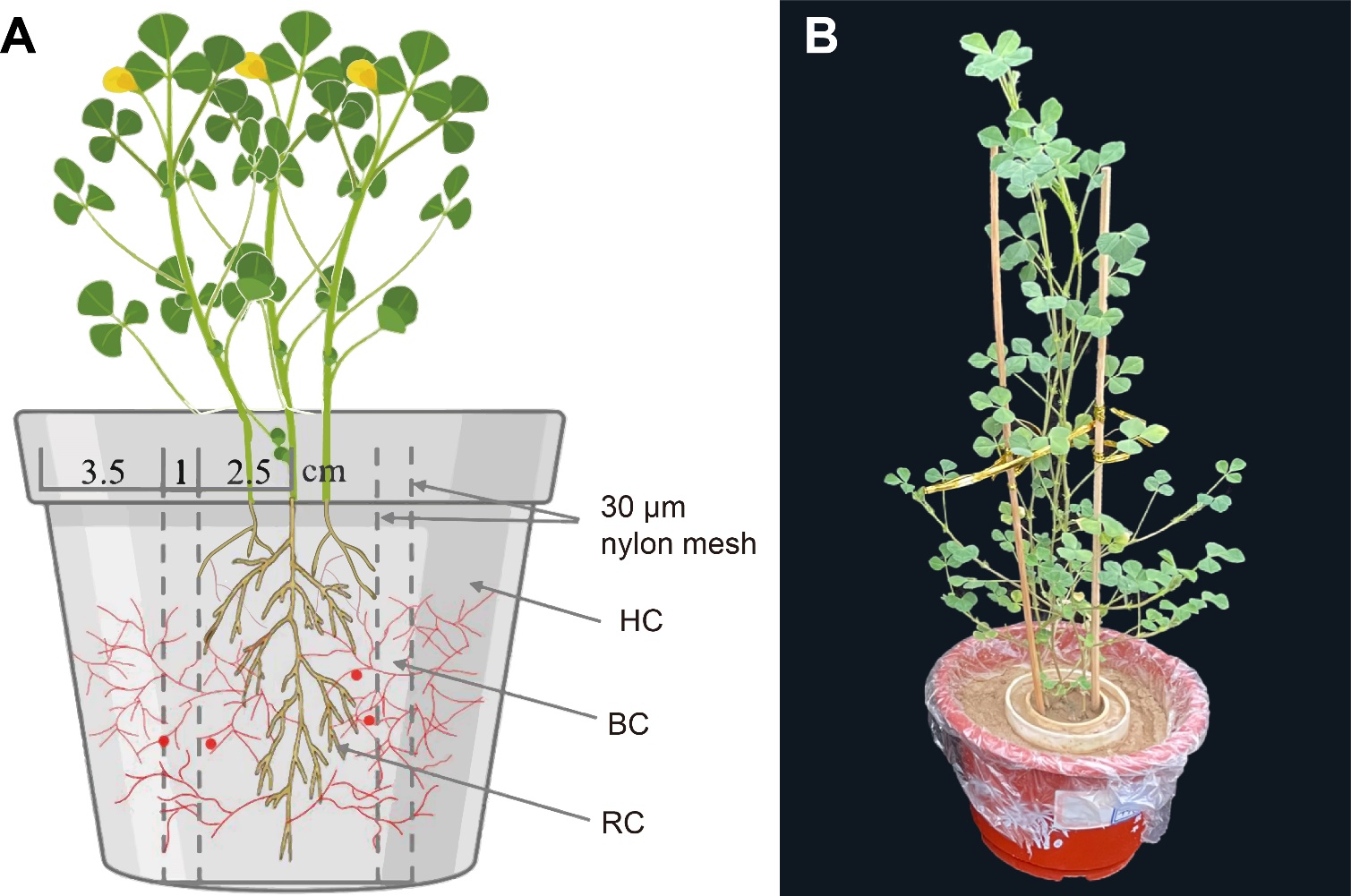
**

**Figure S6** (**A**) Schematic representation of the three-compartmented pot experimental system. The inner compartment contains the roots and fungal hyphae (RC), the middle compartment is a buffer compartment (BC) and the outer compartment is the hyphal compartment (HC) allowing only the extraradical hyphae (ERH) to extend. The BC, with a width of 1 cm, was placed between the two compartments to prevent the movement of soluble organic P from HC to RC. A nylon mesh of 30 μm separating the RC from the BC and the BC from the HC was used to avoid roots to extend from RC to HC, while allowing the hyphae to extend from RC to HC. The bacterium was inoculated in the HC in contact with the ERH of *Rhizophagus irregularis* MUCL 43194. Each treatment consisted of four replicates with three *Medicago truncatula* plants per RC. Red lines, extraradical hyphae of *R. irregularis* MUCL 43194; red dots, spores of *R. irregularis* MUCL 43194. (**B**) Detailed picture of the three-compartmented pot system.

**Tables**

**Table S1** Mean relative abundance of 20 bacterial isolates of all hyphal samples.

| Query_ID | Subject_ID | Identities (%) | Relative abundance (%) |
| --- | --- | --- | --- |
| *Streptomyces* sp. D1 | ASV 8426 | 100 | 10.01 |
| *Nocardioides* *albus* G11 | ASV 7667 | 99 | 0.38 |
| *Mitsuaria* sp. D12 | ASV 6390 | 99 | 0.19 |
| *Pseudomonas* sp. H2 | ASV 10784 | 100 | 0.17 |
| *Stenotrophomonas* *geniculata* H6 | ASV 9430 | 100 | 0.16 |
| *Allo-Neo-Para-Rhizobium* sp. H3 | ASV 5463 | 100 | 0.15 |
| *Chitinophaga* sp. B2 | ASV 1076 | 100 | 0.14 |
| *Stenotrophomonas* *retroflexus* 13 | ASV 7125 | 100 | 0.075 |
| *Lysobacter* *soil* H4 | ASV 12867 | 100 | 0.056 |
| Ensifer sp. E3 | ASV 7924 | 99 | 0.047 |
| *Chitinophaga* sp. 51 | ASV 18452 | 99 | 0.035 |
| *Enterobacter* sp. 44 | ASV 18203 | 100 | 0.015 |
| *Paenarthrobacter* sp. 31 | ASV 8887 | 100 | 0.013 |
| *Flavobacterium* *anhuiense* 14 | ASV 2472 | 100 | 0.011 |
| *Variovorax* sp. H7 | ASV 15874 | 100 | 0.011 |
| *Variovorax* *paradoxus* 6 | ASV 8470 | 100 | 0.0098 |
| *Phyllobacterium* *ifriqiyense* 49 | ASV 9561 | 100 | 0.0090 |
| *Achromobacter* sp. 19 | ASV 18310 | 100 | 0.0013 |
| *Pseudorhodoferax* *soli* 20 | ASV 5009 | 100 | 0.00053 |
| *Chryseobacterium* sp. 22 | ASV 66 | 97 | 0.000089 |

**Table S2** Trehalose metabolism and transporter genes in the genome of *Streptomyces* sp. D1 identified with KEGG.

| Category | Gene | Genes ID | Function descriptions | KO | Start site | End site | Direction | Length |
| --- | --- | --- | --- | --- | --- | --- | --- | --- |
| Trehalose metabolism | *OtsB* | chr_4645 | trehalose 6-phosphate phosphatase | K01087 | 5432754 | 5433626 | + | 873 |
|  | *TREH* | chr_7970 | alpha,alpha-trehalase | K01194 | 9164422 | 9166845 | + | 2424 |
|  | *TREH* | chr_8664 | alpha,alpha-trehalase | K01194 | 10001223 | 10003610 | - | 2388 |
|  | *TreZ* | chr_1924 | maltooligosyltrehalose trehalohydrolase | K01236 | 2350775 | 2352520 | + | 1746 |
|  | *TreS* | chr_2690 | maltose alpha-D-glucosyltransferase / alpha-amylase | K05343 | 3294788 | 3296488 | + | 1701 |
|  | *TreS* | chr_8129 | maltose alpha-D-glucosyltransferase / alpha-amylase | K05343 | 9343599 | 9345317 | - | 1719 |
| Trehalose transporters | *ThuE* | chr_1168 | trehalose/maltose transport system substrate-binding protein | K10236 | 1490313 | 1491533 | - | 1221 |
|  | *ThuF* | chr_1167 | trehalose/maltose transport system permease protein | K10237 | 1489283 | 1490242 | - | 960 |
|  | *ThuG* | chr_1166 | trehalose/maltose transport system permease protein | K10238 | 1488450 | 1489283 | - | 834 |
|  | *MalK* | chr_1165 | multiple sugar transport system ATP-binding protein | K10112 | 1487178 | 1488359 | - | 1182 |

**Table S3** Phosphate metabolism genes in the genome of *Streptomyces* sp. D1 based on KEGG gene annotations.

| Category | Gene | Genes ID | Function descriptions | KO | Start site | End site | Direction | Length |
| --- | --- | --- | --- | --- | --- | --- | --- | --- |
| organic P mineralization | *phy* | chr_1005 | 3-phytase | K01083 | 1303238 | 1304551 | - | 1314 |
|  | *phoD* | chr_6122 | alkaline phosphatase D | K01113 | 7129151 | 7130791 | + | 1641 |
|  | *phoD* | chr_7332 | alkaline phosphatase D | K01113 | 8456816 | 8458390 | - | 1575 |
|  | *phoX* | chr_4153 | alkaline phosphatase | K07093 | 4890514 | 4892484 | - | 1971 |
|  | *glpQ* | chr_4341 | glycerophosphoryl diester phosphodiesterase (periplasmic) | K01126 | 5098529 | 5099377 | - | 849 |
|  | *glpQ* | chr_6488 | glycerophosphoryl diester phosphodiesterase (periplasmic) | K01126 | 7530407 | 7531276 | - | 870 |
|  | *glpQ* | chr_7017 | glycerophosphoryl diester phosphodiesterase (periplasmic) | K01126 | 8106089 | 8107252 | + | 1164 |
|  | *glpQ* | chr_7176 | glycerophosphoryl diester phosphodiesterase (periplasmic) | K01126 | 8276599 | 8277369 | - | 771 |
|  | *glpQ* | chr_7603 | glycerophosphoryl diester phosphodiesterase (periplasmic) | K01126 | 8770425 | 8771075 | - | 651 |
|  | *opd* | chr_7711 | phosphotriesterase (organophosphorus-degrading genes) | K07048 | 8883657 | 8884565 | - | 909 |
| Inorganic P solubilization | *gdh* | chr_5861 | glucose 1-dehydrogenase | K00034 | 6835502 | 6836341 | - | 840 |
|  | *ppa* | chr_3773 | inorganic pyrophosphatase | K01507 | 4495354 | 4495845 | + | 492 |
|  | *ppx* | chr_3717 | exopolyphosphatase / guanosine-5'-triphosphate,3'-diphosphate pyrophosphatase | K01524 | 4433323 | 4434255 | - | 933 |
|  | *ppx* | chr_4079 | exopolyphosphatase / guanosine-5'-triphosphate,4'-diphosphate pyrophosphatase | K01524 | 4818274 | 4819290 | + | 1017 |
|  | *ppx* | chr_5051 | exopolyphosphatase / guanosine-5'-triphosphate,5'-diphosphate pyrophosphatase | K01524 | 5896053 | 5897018 | + | 966 |
|  | *ppk2* | chr_7972 | polyphosphate kinase | K22468 | 9168051 | 9168854 | - | 804 |
|  | *ppk2* | chr_906 | polyphosphate kinase | K22468 | 1022452 | 1023345 | - | 894 |
|  | *ppk* | chr_4496 | polyphosphate kinase | K00937 | 5284889 | 5287126 | - | 2238 |
|  | *ppa* | chr_3773 | inorganic pyrophosphatase | K01507 | 4495354 | 4495845 | + | 492 |
| Transporters | *pstS* | chr_4493 | phosphate transport system substrate-binding protein | K02040 | 5281879 | 5283012 | - | 1134 |
|  | *pstS* | chr_4676 | phosphate transport system substrate-binding protein | K02040 | 5465817 | 5467457 | + | 1641 |
|  | *pstC* | chr_4492 | phosphate transport system permease protein | K02037 | 5280777 | 5281775 | - | 999 |
|  | *pstB* | chr_4490 | phosphate transport system ATP-binding protein | K02036 | 5278891 | 5279667 | - | 777 |
|  | *TC.PIT* | chr_4485 | inorganic phosphate transporter, PiT family | K03306 | 5275052 | 5276050 | - | 999 |
|  | *TC.PIT* | chr_6661 | inorganic phosphate transporter, PiT family | K03306 | 7716176 | 7717411 | - | 1236 |
|  | *ugpB* | chr_5568 | sn-glycerol 3-phosphate transport system substrate-binding protein | K05813 | 6482883 | 6484244 | - | 1362 |
|  | *ugpA* | chr_639 | sn-glycerol 3-phosphate transport system permease protein | K05814 | 726291 | 727181 | - | 891 |
|  | *ugpE* | chr_1380 | sn-glycerol 3-phosphate transport system permease protein | K05815 | 1727896 | 1728735 | + | 840 |
|  | *phnS* | chr_5329 | 2-aminoethylphosphonate transport system substrate-binding protein | K11081 | 6221118 | 6222173 | + | 1056 |
|  | *phnV* | chr_5328 | 2-aminoethylphosphonate transport system permease protein | K11082 | 6220408 | 6221079 | + | 672 |
|  | *phnU* | chr_5327 | 2-aminoethylphosphonate transport system permease protein | K11083 | 6219528 | 6220295 | + | 768 |
|  | *phnT* | chr_5326 | 2-aminoethylphosphonate transport system ATP-binding protein | K11084 | 6218381 | 6219442 | + | 1062 |
| Regulatory genes | *phoU* | chr_4585 | phosphate transport system protein | K02039 | 5371178 | 5371879 | - | 702 |
|  | *phoR* | chr_4586 | two-component system, OmpR family, phosphate regulon sensor histidine kinase PhoR | K07636 | 5372337 | 5373614 | + | 1278 |

**Table S4** Phosphate metabolism genes in the genome of *Pseudomonas* sp. H2 based on KEGG gene annotations.

| Category | Genes names | Genes ID | Function descriptions | KO | Start site | End site | Direction | Length |
| --- | --- | --- | --- | --- | --- | --- | --- | --- |
| organic P mineralization | *phy* | chr_2595 | 3-phytase | K01083 | 2832466 | 2834379 | + | 1914 |
|  | *phoD* | chr_997 | alkaline phosphatase D | K01113 | 1155990 | 1157531 | + | 1542 |
|  | *phoX* | chr_5395 | alkaline phosphatase | K07093 | 5976401 | 5978302 | + | 1902 |
|  | *glpQ* | chr_1626 | glycerophosphoryl diester phosphodiesterase (periplasmic) | K01126 | 1810264 | 1811391 | - | 1128 |
|  | *glpQ* | chr_2166 | glycerophosphoryl diester phosphodiesterase (periplasmic) | K01126 | 2406282 | 2407004 | - | 723 |
|  | *phnX* | chr_4073 | phosphonoacetaldehyde hydrolase | K05306 | 4504295 | 4505122 | + | 828 |
|  | *phnW* | chr_4072 | 2-aminoethylphosphonate-pyruvate transaminase | K03430 | 4503031 | 4504140 | + | 1110 |
| Inorganic P solubilization | *gcd* | chr_4776 | quinoprotein glucose dehydrogenase | K00117 | 5306293 | 5308701 | + | 2409 |
|  | *ppa* | chr_5210 | inorganic pyrophosphatase | K01507 | 5781892 | 5782419 | + | 528 |
|  | *ppx* | chr_5672 | exopolyphosphatase / guanosine-5'-triphosphate,3'-diphosphate pyrophosphatase | K01524 | 6276765 | 6278267 | + | 1503 |
| Transporters | *pstA* | chr_130 | phosphate transport system permease protein | K02038 | 139842 | 141512 | - | 1671 |
|  | *pstC* | chr_131 | phosphate transport system permease protein | K02037 | 141528 | 143810 | - | 2283 |
|  | *pstS* | chr_132 | phosphate transport system substrate-binding protein | K02040 | 144046 | 145044 | - | 999 |
|  | *pstS* | chr_4038 | phosphate transport system substrate-binding protein | K02040 | 4465041 | 4466381 | + | 1341 |
|  | *pstB* | chr_129 | phosphate transport system ATP-binding protein | K02036 | 138885 | 139718 | - | 834 |
|  | *phnD* | chr_2587 | phosphonate transport system substrate-binding protein | K02044 | 2823178 | 2824029 | - | 852 |
|  | *phnC* | chr_2586 | phosphonate transport system ATP-binding protein | K02041 | 2822384 | 2823181 | - | 798 |
|  | *phnE* | chr_2584 | phosphonate transport system permease protein | K02042 | 2820799 | 2821566 | - | 768 |
|  | *phnE* | chr_2585 | phosphonate transport system permease protein | K02042 | 2821563 | 2822336 | - | 774 |
| Regulatory genes | *phoB* | chr_123 | two-component system, OmpR family, phosphate regulon response regulator PhoB | K07657 | 132181 | 132870 | + | 690 |
|  | *phoR* | chr_124 | two-component system, OmpR family, phosphate regulon sensor histidine kinase PhoR | K07636 | 132909 | 134231 | + | 1323 |
|  | *phoU* | chr_128 | phosphate transport system protein | K02039 | 138052 | 138813 | - | 762 |

**Table S5** Number of genes significantly differentially expressed for each KEGG metabolic pathways in *Pseudomonas* sp. H2.

| KEGG pathway | DEG number | Up number | Down number | Total number |
| --- | --- | --- | --- | --- |
| Carbohydrate metabolism | 160 | 151 | 9 | 482 |
| Amino acid metabolism | 157 | 157 | 0 | 453 |
| Energy metabolism | 93 | 88 | 5 | 226 |
| Translation | 72 | 72 | 0 | 79 |
| Metabolism of cofactors and vitamins | 60 | 60 | 0 | 219 |
| Membrane transport | 52 | 51 | 1 | 276 |
| Nucleotide metabolism | 46 | 46 | 0 | 94 |
| Cellular community - prokaryotes | 37 | 36 | 1 | 135 |
| Lipid metabolism | 35 | 34 | 1 | 109 |
| Signal transduction | 33 | 32 | 1 | 210 |
| Metabolism of other amino acids | 31 | 30 | 1 | 105 |
| Folding, sorting and degradation | 27 | 27 | 0 | 54 |
| Xenobiotics biodegradation and metabolism | 26 | 26 | 0 | 80 |
| Metabolism of terpenoids and polyketides | 23 | 23 | 0 | 55 |
| Replication and repair | 22 | 20 | 2 | 81 |
| Glycan biosynthesis and metabolism | 21 | 18 | 3 | 83 |
| Biosynthesis of other secondary metabolites | 20 | 20 | 0 | 47 |
| Cell motility | 17 | 17 | 0 | 89 |
| Infectious disease: bacterial | 7 | 7 | 0 | 33 |
| Drug resistance: antimicrobial | 6 | 6 | 0 | 49 |
| Cell growth and death | 5 | 5 | 0 | 15 |
| Environmental adaptation | 3 | 3 | 0 | 7 |
| Transcription | 3 | 3 | 0 | 4 |
| Immune system | 1 | 1 | 0 | 3 |

**Table S6** Secondary metabolites gene clusters in the genome of *Streptomyces* sp. D1 detected by antiSMASH6 pipeline.

| Region | Type | From | To | Most similar known cluster | Similarity |
| --- | --- | --- | --- | --- | --- |
| Region 1 | T1PKS | 124,395 | 164,066 | 4-hexadecanoyl-3-hydroxy-2-(hydroxymethyl)-2H-furan-5-one | 100% |
| Region 2 | T3PKS,NRPS | 382,638 | 476,235 | isocomplestatin | 100% |
| Region 3 | RiPP-like | 552,566 | 562,641 |  |  |
| Region 4 | NRPS,T1PKS | 657,579 | 707,196 |  |  |
| Region 5 | siderophore | 762,084 | 774,287 |  |  |
| Region 6 | lanthipeptide-class-iii,RiPP-like | 793,348 | 819,013 | informatipeptin | 100% |
| Region 7 | T1PKS | 1,041,089 | 1,275,716 | nystatin A1 | 45% |
| Region 8 | terpene | 1,561,139 | 1,585,578 | hopene | 92% |
| Region 9 | redox-cofactor | 1,886,952 | 1,907,656 |  |  |
| Region 10 | lassopeptide | 2,064,551 | 2,087,106 | citrulassin D | 100% |
| Region 11 | siderophore | 2,113,645 | 2,125,935 | grincamycin | 8% |
| Region 12 | NAPAA | 2,164,977 | 2,198,893 | stenothricin | 13% |
| Region 13 | terpene, butyrolactone | 2,350,039 | 2,374,349 | γ-butyrolactone | 100% |
| Region 14 | RiPP-like | 2,464,188 | 2,475,504 |  |  |
| Region 15 | betalactone | 2,545,876 | 2,571,267 | vazabitide A | 6% |
| Region 16 | T1PKS | 2,593,494 | 2,653,911 | argimycin PI / argimycin PII / nigrifactin / argimycin PIV / argimycin PV / argimycin PVI / argimycin PIX | 24% |
| Region 17 | siderophore | 2,819,359 | 2,829,691 |  |  |
| Region 18 | T2PKS | 3,303,166 | 3,373,730 | spore pigment | 75% |
| Region 19 | NRPS | 3,391,400 | 3,447,602 | arginomycin | 20% |
| Region 20 | terpene | 3,521,575 | 3,538,481 | albaflavenone | 100% |
| Region 21 | redox-cofactor | 4,179,984 | 4,202,117 | lankacidin C | 13% |
| Region 22 | CDPS | 4,615,903 | 4,636,658 |  |  |
| Region 23 | thioamide-NRP,NRPS, ladderane,T2PKS | 5,622,991 | 5,742,897 | ishigamide | 100% |
| Region 24 | siderophore | 6,282,130 | 6,293,902 | desferrioxamin B / desferrioxamine E | 83% |
| Region 25 | melanin, butyrolactone | 6,392,737 | 6,407,035 | istamycin | 4% |
| Region 26 | indole | 7,016,562 | 7,036,297 | 5-isoprenylindole-3-carboxylate β-D-glycosyl ester | 28% |
| Region 27 | ectoine | 7,684,035 | 7,694,439 | ectoine | 100% |
| Region 28 | NAPAA | 7,928,384 | 7,962,238 |  |  |
| Region 29 | terpene | 8,013,469 | 8,031,723 | pradimicin-A | 7% |
| Region 30 | ladderane | 8,797,034 | 8,838,299 | foxicins A-D | 24% |
| Region 31 | terpene, melanin | 9,267,096 | 9,288,959 | melanin | 57% |
| Region 32 | T1PKS,NRPS | 9,369,899 | 9,485,898 | coelichelin | 100% |
| Region 33 | T1PKS | 9,491,406 | 9,539,295 | lagunapyrone A / lagunapyrone B / lagunapyrone C | 22% |
| Region 34 | indole | 9,883,630 | 9,905,045 | salinomycin | 10% |

**Table S7** Physico-chemical properties of the soils used to extract bacterial suspensions.

| Soil ID | Soil types | pH | Total P  (g kg^−1^) | Total N  (g kg^−1^) | NaHCO_3_ extractable P (mg kg^−1^) | NH_4_OAC extractable K (mg kg^−1^) | SOC  (g kg^−1^) |
| --- | --- | --- | --- | --- | --- | --- | --- |
| soil SH | Gray desert soil | 8.03 | 1.12 | 0.97 | 70.0 | 436 | 9.51 |
| soil BJ | Moisture soil | 8.20 | 0.56 | 0.80 | 58.90 | 94.10 | 14.74 |
| soil QY | Red soil | 5.70 | 1.03 | 1.07 | 13.90 | 122 | 11.50 |
| soil WL | Gray desert soil | 7.99 | 0.94 | 1.06 | 11.50 | 206 | 9.47 |
| soil TA | Brown soil | 6.51 | NA | NA | 3.60 | 37.6 | 3.40 |
| soil Changping, BJ | Moisture soil | 8.35 | NA | 1.35 | 3.80 | 214.2 | 21.53 |

Note: SOC, soil organic carbon.

**Table S8** Summary of primers used in the study.

| Target region | Forward primer | Forward Primer sequence | Reverse Primer | Reverse Primer sequence |
| --- | --- | --- | --- | --- |
| 16S rRNA genes | 338F | ACTCCTACGGGAGGCAGCA | 806R | GGACTACHVGGGTWTCTAAT |
| *St* | StF | TCCTGGGTGGAGGTTAAAAG | StR | GTGCAATATTCCCCACTGCT |
| *Ps* | PsF | GGAATCTGCCTGGTAGTGGG | PsR | GTCTGGACCGTGTCTCAGTT |
| *5.8s rRNA* | 5.8s-F | GTATGCCTGTTTGAGGGTCAGTATT | 5.8s-R | AAACTCCGGAACGTCACTAAAGAG |
| *Pho84* | Pho84-F | CAAACGCATTTGTTGGAC | Pho84-R | GCTTCCGCCTTTACTCTT |
| *Pho91* | Pho91-F | GAGCAGTGATGATGACGATGA | Pho91-R | CTGTTCCTCAAGTGACGATGG |
| *VTC2* | VTC2-F | GTCGAAGTGGTGGGCGGTAG | VTC2-R | ATCGGTCTTTGTTGTTCATCCTCA |
| *VTC4* | VTC4-F | CCATTCCTGTGCGTGTTG | VTC4-R | CCGCCATCGTGATAGTAGTTAGT |
| *RiPT7* | RiPT7-F | AGATGGTGCAAGAAATTCAACTG | RiPT7-R | AGTTCTGACAACCAAACGAAAAG |
| *MtPT4* | MtPT4-F | GACACGAGGCGCTTTCATAGCAGC | MtPT4-R | GTCATCGCAGCTGGAACAGCACCG |
| *MtHA1* | MtHA1-F | GTTTGTTGCCCTTCTCCCTCTTTT | MtHA1-R | GCCTTCCCGTTTCCTTTCCTAT |
| *FatM* | FatM-F | TTGAGCAAAGGCCAATAAGGT | FatM-R | CTATGTAGAAAATGGACATGTAGTGA |
| *RAM2* | RAM2-F | TTGGTGATGAAAAGCCTGAT | RAM2-R | AAGATTATGGGTTTTGGAAGTTTG |
| *WRI5a* | WRI5a-F | AGAGGAGTAGCAAGGCACCATC | WRI5a-R | AGGTCCTGGCCTTAACCATCTT |
| *WRI5b* | WRI5b-F | TGTACCAAAAATAGGTGATGATGCT | WRI5b-R | TCCATCTATGCCTGCTAACACC |
| *WRI5c* | WRI5c-F | GCACCACCACAACGGTAGAT | WRI5c-R | TCCTCTTGGGTGCTGTAGGT |
| *RAM1* | RAM1-F | CTCATTCTCTTCGTATCCCCTTT | RAM1-R | GTTTGGTGCTTGGTCTCTTATCAT |
| *MtEF-1* | MtEF-1-F | CTTTGCTTGGTGCTGTTTAGATGG | MtEF-1-R | ATTCCAAAGGCGGCTGCATA |

**Table S9** Phosphate, polyphosphate and associated genes expression significantly increased in *Rhizophagus* *irregularis* MUCL 43194 inoculated with *Streptomyces* sp. D1 as compared to the fungus grown in absence of the bacteria.

| Category | Genes | Gene ID | Function descriptions | Start Site | End Site | Direction | Length |
| --- | --- | --- | --- | --- | --- | --- | --- |
| phosphate-associated genes | *pho80* | GLOIN_2v1555064 | High-affinity H+/Pi cotransporter | 152971 | 155036 | + | 1900 |
|  | *pho89* | GLOIN_2v1589937 | Na+/Pi cotransporter | 18309 | 20435 | + | 1821 |
|  | *VTC1* | GLOIN_2v1540144 | Regulatory subunit of vacuolar transporter chaperone (VTC) complex | 269186 | 270404 | + | 981 |
|  | *VTC4* | GLOIN_2v1578147 | Regulatory subunit of vacuolar transporter chaperone (VTC) complex | 39321 | 42110 | - | 2651 |
|  | *VTC2* | GLOIN_2v1593766 | Regulatory subunit of vacuolar transporter chaperone (VTC) complex | 163463 | 166270 | + | 2496 |
|  | *pho4* | GLOIN_2v1602893 | activates transcription response to phosphate limitation | 44730 | 47265 | - | 1988 |
|  | *pho80* | GLOIN_2v1465109 | regulates the response phosphate limitation | 68236 | 68989 | + | 612 |
|  | *pho85* | GLOIN_2v1641429 | regulating the cellular response to nutrient levels | 135470 | 138915 | - | 1049 |
|  | *pho85* | GLOIN_2v1689057 | regulating the cellular response to nutrient levels | 13527 | 14787 | + | 1013 |
|  | *pho85* | GLOIN_2v1733465 | regulating the cellular response to nutrient levels | 19676 | 22915 | + | 3082 |
|  | *pho85* | GLOIN_2v1877748 | regulating the cellular response to nutrient levels | 63091 | 65423 | - | 2015 |
|  | *pho85* | GLOIN_2v1453641 | regulating the cellular response to nutrient levels | 430525 | 431857 | - | 1236 |
|  | *pho85* | GLOIN_2v1474244 | regulating the cellular response to nutrient levels | 381406 | 383611 | + | 1365 |
|  | *pho85* | GLOIN_2v1558917 | regulating the cellular response to nutrient levels | 267973 | 269928 | + | 1363 |
|  | *pho85* | GLOIN_2v1578138 | regulating the cellular response to nutrient levels | 25745 | 27131 | + | 1103 |
|  | *pho81* | GLOIN_2v1676302 | regulates Pho80-Pho85 cyclin-CDK complexes in response to phosphate levels | 23538 | 27915 | - | 3784 |
|  | *pho91* | GLOIN_2v1675531 | exports phosphate from vacuolar lumen to cytosol | 146706 | 150188 | - | 3031 |
|  | *ppn1* | GLOIN_2v1134418 | acts as both an endopolyphosphatase cleaving long chains of polyphosphate distributively to generate shorter polymer chains and as an exopolyphosphatase catalyzing the hydrolysis of terminal phosphate from polyphosphate | 270254 | 272782 | + | 2419 |
|  | *ppn1* | GLOIN_2v1594422 | acts as both an endopolyphosphatase cleaving long chains of polyphosphate distributively to generate shorter polymer chains and as an exopolyphosphatase catalyzing the hydrolysis of terminal phosphate from polyphosphate | 88796 | 90996 | + | 1753 |
|  | *ppn1* | GLOIN_2v1778724 | acts as both an endopolyphosphatase cleaving long chains of polyphosphate distributively to generate shorter polymer chains and as an exopolyphosphatase catalyzing the hydrolysis of terminal phosphate from polyphosphate | 86419 | 88212 | + | 1677 |
| metal ion transporter | *VIT1* | GLOIN_2v1456385 | vacuolar iron transporter 1 | 111280 | 112350 | + | 822 |
|  | *ZRT1* | GLOIN_2v1741254 | high-affinity zinc transporter of the plasma membrane | 315624 | 317064 | + | 1092 |
|  | *MatA* | GLOIN_2v1727187 | magnesium transporting ATPase | 47551 | 51283 | + | 3240 |
| energy production | *Idh2* | GLOIN_2v1686949 | Isocitrate dehydrogenase [NADP] | 135126 | 137962 | - | 1593 |
|  | *NDUFA4* | GLOIN_2v1595142 | transfer electrons from NADH to the respiratory chain | 49879 | 50822 | - | 649 |
|  | *ssuE* | GLOIN_2v1591466 | reduces FMN to FMNH2 in a NAD(P)H-dependent manner | 117741 | 118796 | - | 758 |
|  | *wrbA* | GLOIN_2v1620358 | NAD(P)H dehydrogenase [quinone] | 132122 | 133125 | - | 820 |

**Reference**

1. Zhang J, Liu YX, Guo X, Qin Y, Garrido-Oter R, Schulze-Lefert P, et al. High-throughput cultivation and identification of bacteria from the plant root microbiota. Nat. protoc. 2021;16:988-1012.

2. Toljander JF, Lindahl BD, Paul LR, Elfstrand M, Finlay RD. Influence of arbuscular mycorrhizal mycelial exudates on soil bacterial growth and community structure. FEMS Microbiol. Ecol. 2007;61:295-304.

3. Bharadwaj DP, Alström S, Lundquist PO. Interactions among *Glomus* *irregulare*, arbuscular mycorrhizal spore-associated bacteria, and plant pathogens under in vitro conditions. Mycorrhiza 2012;22:437-447.

4. Luthfiana N, Inamura N, Tantriani, Sato T, Saito K, Oikawa A, et al. Metabolite profiling of the hyphal exudates of *Rhizophagus* *clarus* and *Rhizophagus* *irregularis* under phosphorus deficiency. Mycorrhiza 2021;31(3):403-412.

5. Elbing KL, Brent R. Media preparation and bacteriological tools. Curr Protoc Protein Sci 2001;Chapter 1:Unit 1.1.

6. Finkel OM, Salas-González I, Castrillo G, Conway JM, Law TF, Teixeira P, et al. A single bacterial genus maintains root growth in a complex microbiome. Nature 2020;587:103-108.

7. Doner LW, Becard G. Solubilization of gellan gels by chelation of cations. Biotechnol. Tech. 1991;5:25-28.

8. Bolyen E, Rideout JR, Dillon MR, Bokulich NA, Abnet CC, Al-Ghalith GA, et al. Reproducible, interactive, scalable and extensible microbiome data science using QIIME 2. *Nat*. *Biotechnol*. 2019;37:852-857.

9. Martin MJEJ. Cutadapt removes adapter sequences from high-throughput sequencing reads. EMBnet j. 2011;17: http://dx.doi.org/10.14806/ej.17.1.200.

10. Callahan BJ, McMurdie PJ, Rosen MJ, Han AW, Johnson AJ, Holmes SP. DADA2: High-resolution sample inference from Illumina amplicon data. Nat. Methods 2016;13:581-583.

11. Katoh K, Misawa K, Kuma K, Miyata T. MAFFT. a novel method for rapid multiple sequence alignment based on fast Fourier transform. *Nucleic* *Acids* *Res*. 2002;30:3059-3066.

12. Bokulich NA, Kaehler BD, Rideout JR, Dillon M, Bolyen E, Knight R, et al. Optimizing taxonomic classification of marker-gene amplicon sequences with QIIME 2's q2-feature-classifier plugin. Microbiome 2018;6:90.

13. Quast C, Pruesse E, Yilmaz P, Gerken J, Schweer T, Yarza P, et al. The SILVA ribosomal RNA gene database project: improved data processing and web-based tools. Nucleic Acids Res. 2013;41:D590-596.
